# A hybrid dual-stream CNN framework with dynamic data augmentation and improved Manta Ray Foraging Optimization for robust glaucoma detection

**DOI:** 10.1038/s41598-026-45384-6

**Published:** 2026-04-17

**Authors:** Azza Atia, Hatem Abdel-kader, Osama M. Abo-Seida, Amira Abdelatey

**Affiliations:** 1 Department of Information Systems, Faculty of Computers and Information, Kafrelsheikh University, Kafrelsheikh, Egypt; 2 Department of Information Systems, Faculty of Computers and Information, Menoufia University, Shibin El-Kom, Al Minufiyah Egypt; 3 Department of Computer Science, Faculty of Computers and Information, Kafrelsheikh University, Kafrelsheikh, Egypt; 4 Faculty of Computers and Artificial Intelligence, Menoufia National University, Tukh Tambisha, Al Minufiyah Egypt

**Keywords:** Glaucoma detection, Lightweight channel-wise attention mechanism, Dual-stream convolutional neural network (CNN), Dynamic Gaussian noise

## Abstract

A progressive neurological condition, glaucoma is one of the main causes of irreversible blindness in the globe. Early detection is crucial to preventing irreversible vision loss however conventional diagnostic methods are often time-consuming, and heavily reliant on clinical expertise. This study presents an innovative deep learning framework designed to automate glaucoma detection while addressing key challenges such as data imbalance, image quality inconsistencies, and the need for accurate feature extraction. The proposed framework begins with a dedicated preprocessing pipeline that enhances image clarity, and isolates clinically relevant regions of interest. To handle class imbalance, a novel hybrid data augmentation strategy is introduced, combining geometric transformations with adaptive Gaussian noise injection that adjusts intensity according to image characteristics, thereby simulating realistic clinical variability. At its core, the framework leverages a dual-stream CNN architecture that integrates DenseNet121 for structural feature extraction and ResNet50 for texture representation. A lightweight channel-wise attention mechanism is then introduced to selectively emphasize clinically significant channels while suppressing redundant features, thereby balancing efficiency and interpretability. To optimize model performance while reducing computational overhead, an Improved Manta Ray Foraging Optimization (IMRFO) algorithm is employed. IMRFO enhances the standard MRFO with Partial Centroid Opposition-Based Learning (PCOBL) to dynamically fine-tune hyperparameters, including augmentation settings and transfer learning configurations. Experimental validation was conducted on four public benchmark datasets, ACRIMA, Drishti-Gs, ORIGA, and RIM-ONE-DL, demonstrating the framework’s superior performance across all evaluation metrics. The model achieved 100.00% accuracy, precision, recall, and AUC on both ACRIMA and Drishti-Gs, with losses of 0.003 and 0.001, respectively. On ORIGA, it reached 99.70% accuracy, 99.80% precision, 99.30% recall, and 99.50% AUC (loss = 0.01). On RIM-ONE-DL, the model scored 99.90% accuracy, 99.70% precision, 99.50% recall, and 99.70% AUC (loss = 0.006). These findings confirm the framework’s robustness and clinical applicability for effective glaucoma screening.

## Introduction

Glaucoma is a degenerative eye condition marked by ongoing harm to the optic disc and retinal ganglion cells, positioning it as the second most common cause of permanent blindness globally^1^. Currently, the disease affects approximately 80 million individuals, with projections estimating over 111 million cases by 2040 and more than 4.5 million instances of blindness annually^2^. While aging and ethnicity are established risk factors, glaucoma spans all demographics and is prevalent in both developed and developing regions. Its asymptomatic progression during early stages often leads to delayed diagnosis; nearly 50% of those affected remain unaware until significant vision loss occurs^3^. However, early intervention can delay blindness by up to 20 years in half of these cases, emphasizing the critical need for timely and accurate diagnosis^4^.

**Fig. 1:**
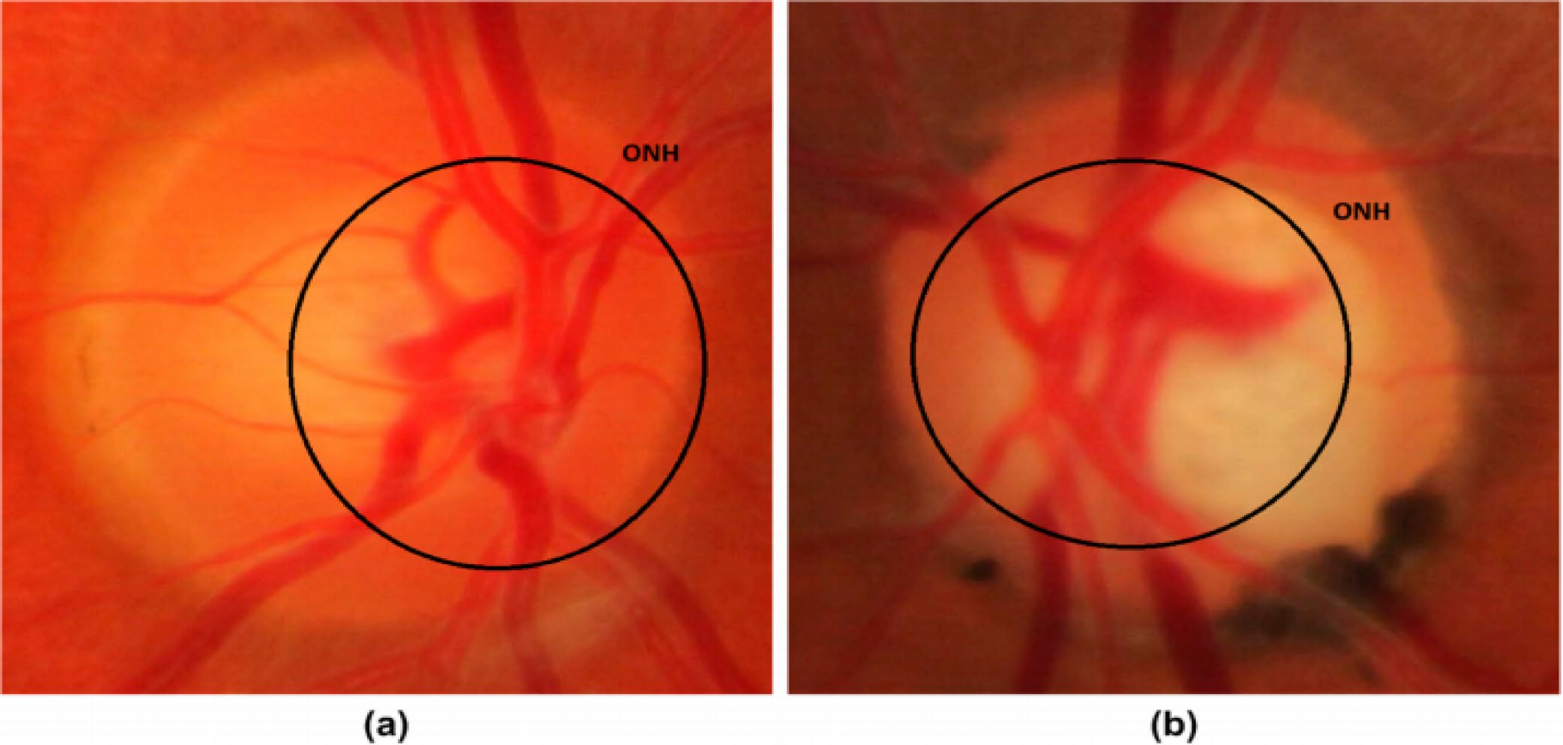
Representative ONH images: (**a**) normal eye and (**b**) glaucoma-affected eye.

Conventional diagnostic methods for glaucoma primarily rely on assessing the optic nerve head (ONH) through techniques such as fundus photography, intraocular pressure (IOP) measurement, and visual field testing. Structural features of the ONH, particularly the optic disc (OD) and optic cup (OC), are key indicators for glaucoma assessment and are commonly analyzed using color fundus photography (CFP) and optical coherence tomography (OCT). Several studies have demonstrated that accurate OD and OC segmentation, as well as cup-to-disc ratio (CDR) estimation from fundus images, play a crucial role in reliable glaucoma classification and disease progression analysis^5,6^. While OCT provides high-resolution cross-sectional views of the retina, CFP remains more widely adopted due to its accessibility and cost-effectiveness. In glaucomatous eyes, elevated IOP often leads to optic cup enlargement and progressive nerve fiber layer damage, which, if untreated, results in irreversible vision loss^7,8^. Figure 1 illustrates these pathological changes, including optic disc deformation and intraocular fluid (IOF) obstruction. Despite their clinical utility, conventional approaches are time-consuming, dependent on expert interpretation, and prone to inter-observer variability. To overcome these limitations, artificial intelligence (AI) particularly deep learning (DL) and convolutional neural networks (CNNs) has emerged as a promising solution for automating glaucoma diagnosis^9^. These models demonstrate high diagnostic accuracy by automatically learning disease-relevant features from CFP and OCT scans, with CNN-based frameworks achieving performance levels comparable to trained specialists^10,11^.

Despite recent advancements in deep learning, the clinical deployment of CNN-based glaucoma detection systems continues to face several critical challenges. Among these are data imbalance, where non-glaucomatous images significantly outnumber glaucomatous ones, leading to biased model predictions; variability in image acquisition conditions such as differences in resolution, brightness, and imaging devices, which adversely affects model generalizability across diverse clinical environments; and domain adaptation limitations, where models trained on specific datasets often exhibit degraded performance when applied to unseen data. Furthermore, many existing models struggle with the precision of feature extraction, , as conventional CNN architectures may overlook subtle yet clinically important structural and textural variations within the optic nerve head. Such fine-grained patterns, including localized optic disc deformation and texture irregularities, are critical for accurate glaucoma diagnosis but are often inadequately captured by single-stream or standard convolutional designs^12,13^. Finally, the high computational demands of state-of-the-art CNN architectures pose a barrier to real-time deployment, particularly in resource-constrained settings. To effectively address the aforementioned challenges, this paper proposes a novel and robust framework for automated glaucoma detection, which provides the following significant contributions:

- **Hybrid Data Augmentation Strategy**: To mitigate data imbalance and enhance generalization under variable imaging conditions, a hybrid augmentation pipeline is proposed. It combines traditional geometric transformations (e.g., rotation, shift, zoom, and shear) with a novel adaptive Gaussian noise injection mechanism. This technique dynamically modulates noise intensity based on the brightness and contrast of each image, effectively simulating real-world clinical noise patterns while preserving diagnostic details.
- **Dual-Stream CNN Architecture for Enhanced Feature Extraction**: To address the challenge of imprecise feature representation in traditional CNNs, the framework introduces dual-stream architecture. DenseNet121 is used to capture structural features of the optic nerve head, while ResNet50 focuses on fine-grained texture information. This dual-pathway design allows the model to learn complementary features essential for accurate glaucoma detection.
- **Lightweight Channel-Wise Attention Mechanism**: To further refine the extracted features and enhance model interpretability, a lightweight channel-wise attention mechanism is integrated into both CNN streams. This mechanism enables the model to selectively focus on clinically relevant channels, by dynamically assigning higher attention weights to discriminative features.
- **Optimized Hyperparameter Selection via IMRFO**: To enhance convergence and reduce computational cost, MRFO with PCOBL (IMRFO) is adopted for hyperparameter tuning. It dynamically optimizes augmentation parameters (rotation, shift, zoom, and shear) and transfer learning settings (learning rate, batch size, optimizer, and trainable layers), ensuring optimal and robust model performance with minimal manual effort.
- **Comprehensive Evaluation**: The proposed framework is validated on four public datasets: ACRIMA, Drishti-Gs, ORIGA, and RIM-ONE-DL. It attains top-tier performance across a variety of metrics (accuracy, precision, recall, AUC, and loss), confirming its robustness, efficiency, and clinical applicability.

The paper is organized into the following sections: Section "Literature review" reviews prior work and highlights existing challenges, Section "Preliminaries" introduces the background concepts, Sect. 4 elaborates on the proposed approach, Section "Experimental results and performance analysis" presents and analyzes the experimental results, and Section "Conclusion and future work" offers concluding remarks along with directions for future research.

## Literature review

This section reviews prior research on glaucoma lesion detection and classification using fundus images. The methodologies employed in glaucoma recognition are broadly categorized into machine learning (ML)-based approaches and deep learning (DL)-based approaches. This section highlights the progression of these approaches over time, emphasizing their strengths, limitations, and contributions to the field of glaucoma diagnosis.

### Machine learning (ML)-based approaches

Machine learning (ML)-based approaches have traditionally relied on handcrafted features combined with conventional classifiers. For instance, Guo et al.^14^ proposed a framework that assessed the vertical cup-to-disc ratio (VCDR) to identify glaucomatous regions in fundus images. Their method initially applied vasculature- and disc-oriented COSFIRE filters for accurate optic disc detection, followed by the use of a generalized matrix learning vector quantization (GMLVQ) classifier to differentiate between optic disc and optic cup regions. Although this approach enhanced detection accuracy, its performance degraded in the presence of noisy samples, suggesting the need for further refinements. Similarly, Shoba et al.^15^ introduced an ML-based method for glaucoma detection that began with image preprocessing and vessel segmentation using Canny Edge Detection (CED), enhanced through morphological operations. Subsequently, Finite Element Modeling (FEM) was employed to extract discriminative features, which were then classified using a support vector machine (SVM). While this approach demonstrated robustness against noise, its applicability to more complex datasets remains unverified. In another study, Pruthi et al.^16^ presented the Glowworm Swarm Optimization (GSO) algorithm for automated optic cup detection in retinal fundus images. Although the algorithm successfully identified glaucomatous regions, it lacked the capability to compute the cup-to-disc ratio (CDR), a crucial parameter for reliable glaucoma diagnosis.

Qureshi et al.^17^ created a framework for detecting glaucomatous lesions that began with the preprocessing and segmentation of the optic disc (OD) and optic cup (OC) through pixel-based thresholding and watershed transformation. The cup-to-disc ratio (CDR) was calculated by dividing the count of cup pixels by the count of disc pixels. Although this method was effective for identifying lesions, it faced challenges with variations in scale and rotation in the input images. Kirar et al.^18^ introduced a glaucoma detection technique that utilized second-stage quasi-bivariate variational mode decomposition (SS-QB-VMD). This technique produced fine sub-band images (SBIs) from the samples, from which features were extracted and subsequently used to train a least-squares support vector machine (LS-SVM). While this framework demonstrated encouraging results in detecting glaucoma, there is a need for enhancements in classification accuracy.

### Deep learning (DL)-based techniques

Deep learning techniques, especially Convolutional Neural Networks (CNNs), represent notable progress in the field. CNNs are engineered to progressively capture complex and abstract visual attributes from images. Recently, CNN-based methodologies have seen widespread use in various biomedical applications, including glaucoma assessment. For example, Martins et al.^19^ presented a streamlined CNN framework for identifying glaucoma. After a preprocessing stage, they employed MobileNetV2 to extract intricate features from the input images and classify them as either healthy or glaucoma-affected. While this approach is computationally efficient, it depends significantly on a large amount of training data. In a similar vein, Shinde et al.^20^ created a deep learning framework that merges the LeNet architecture for identifying Regions of Interest (RoI) and U-Net for segmenting the optic disc (OD) and optic cup (OC). Classification was executed using support vector machines (SVMs), neural networks (NNs), and AdaBoost classifiers. Although combining results from these classifiers enhanced accuracy, it also led to higher computational demands.

Song et al.^21^ utilized a CNN framework alongside a design-of-experiments (DOE) analysis to effectively tune hyperparameters. Although it demonstrated impressive performance in glaucoma classification, the framework did not undergo testing on standard datasets. Nazir et al.^22^ presented a Fast Region-based Convolutional Neural Network (FRCNN) integrated with fuzzy k-means (FKM) clustering, achieving effective segmentation but incurring significant computational costs. In a separate study, Nazir et al.^23^ applied Mask R-CNN with a DenseNet-77 backbone for segmenting OD and OC lesions in fundus images. While this method produced encouraging results, further improvements are required for optimal performance.

Additional research focused on ensemble techniques and innovative methods for feature extraction. Serte et al.^24^ developed an ensemble model that combined deep features from AlexNet, ResNet-50, and ResNet-152, leading to better classification of normal and glaucoma-impacted regions. Despite the enhanced performance, this approach was also computationally intensive. Nayak et al.^25^ employed the Evolutionary Convolutional Network (ECNet) for the automated classification of glaucoma, extracting key points that were classified using various machine learning techniques (e.g., K-nearest neighbor, SVM, backpropagation neural networks, and extreme learning machines). Even though the SVM classifier produced the most favorable outcomes, it was associated with increased computational demands.

Sreng et al.^26^ developed a multi-phase pipeline that integrates DeepLabV3+ for the segmentation of the optic disc, in conjunction with an SVM and pre-trained CNNs for classification tasks. Although their method achieved high accuracy, it resulted in prolonged training durations. In a similar vein, Elangovan et al.^27^ designed an 18-layer CNN utilizing datasets such as ORIGA and RIM-ONE2, but the random initialization of weight distributions led to inconsistent performance outcomes. In^28^, Chaudhary et al. proposed a boundary detection technique based on a two-dimensional Fourier Bessel series expansion (2D-FBSE-EWT), which decomposed fundus images into sub-images for glaucoma detection by utilizing both traditional machine learning approaches and an ensemble of ResNet-50 models. Nevertheless, this method can be computationally demanding, which constrains its feasibility for real-time applications and in low-resource environments. Gheisari et al.^29^ combined CNNs with LSTM RNNs to extract both spatial and temporal features, achieving a high F-measure; however, the racial homogeneity of their dataset affected its generalizability. In a study conducted by Carvalho et al., a three-dimensional convolutional neural network (3D-CNN) was utilized^30^ to differentiate between glaucomatous and healthy fundus images. This approach involved converting two-dimensional images into three-dimensional volumes for analysis, which attained an accuracy of 96.4%. It is crucial to mention that the conversion of 2D images into 3D volumes may introduce artifacts or cause the loss of essential information, potentially compromising the diagnostic accuracy. Lin et al. introduced GlaucomaNet in^31^, which comprises two CNNs: one dedicated to preliminary grading and the other for more detailed grading. A limitation of GlaucomaNet is its dependence on only two datasets (OHTS and LAG) for assessment, which may limit its generalizability to a wider array of populations and clinical circumstances.

In a study conducted by Kashyap et al.^32^, a method that leveraged U-Net was proposed for data segmentation, while DenseNet-201 was utilized for extracting features. A deep convolutional neural network (DCNN) was employed to classify glaucoma based on retinal fundus images by focusing on the optic cup area and comparing it to the ground truth. Nonetheless, the model’s reliance on a particular dataset may restrict its applicability to different populations and clinical scenarios. In another study, Nawaz et al. presented a model in^33^ that used the EfficientNet-B0 feature extractor, where the extracted features were processed through a Bi-directional Feature Pyramid Network for integrating multiple key points. This model subsequently determined both the location and classification of the glaucoma lesion. The model’s accuracy might be influenced by the variations and resemblances between glaucomatous lesions and natural eye colors, which could lead to possible misclassifications.

Saha et al.^34^ presented a two-step approach for glaucoma detection in fundus images, using a simplified YOLO to locate the optic nerve head (ONH), followed by classification with MobileNetV3small CNN. The system’s accuracy relies on precise ONH localization, as errors in this stage could lead to misclassifications. Fan et al. investigated the explainability and generalizability of Vision Transformer deep learning techniques for identifying glaucoma in^35^, comparing the Data-efficient image Transformer (DeiT) with ResNet-50. While this study evaluated explainability through attention and saliency maps, these visualizations might not be easily interpretable by clinicians. In their study, Shoukat et al. developed a method for glaucoma diagnosis using a ResNet model as described in^36^. Their approach involved utilizing the gray channels of fundus images and implementing data augmentation to train a ResNet-50 model for glaucoma detection. The G1020 dataset demonstrated impressive performance with 98.48% accuracy, 99.30% sensitivity, 96.52% specificity, 97% AUC, and a 98% F1 score. Velpula et al. introduced a model for classifying glaucoma that integrates five pre-trained CNNs: ResNet-50, AlexNet, VGG16, DenseNet-201, and Inception-ResNet-v2^37^. The model employs a basic voting mechanism to determine the final classification, which may not effectively leverage the unique strengths of each CNN. In^38^, Muduli et al. proposed an enhanced computer-aided diagnosis (CAD) framework for automated glaucoma detection, integrating Fast Discrete Curvelet Transform with Wrapping (FDCT-WRP) for feature extraction, Principal Component Analysis (PCA) and Linear Discriminant Analysis (LDA) for dimensionality reduction, and an Extreme Learning Machine (ELM) classifier optimized with the Modified Pelican Optimization Algorithm (MOD-POA) to enhance accuracy and efficiency. The model was evaluated on the G1020 and ORIGA datasets, achieving 93.25% and 96.75% accuracy, respectively, using 10 × 5-fold stratified cross-validation. To improve interpretability, seven XAI techniques were incorporated. While the framework demonstrates high accuracy, efficiency, and transparency, its limitations include dataset diversity constraints, lack of multimodal integration, and the need for further clinical validation.

Subha et al.^39^ introduced a computer-assisted diagnosis (CAD) system for the automatic detection of glaucoma utilizing an ensemble-based deep learning strategy. This system combines three modified pre-trained convolutional neural networks (CNNs), namely ResNet50, VGGNet19, and Inception-V3, to derive structural features from segmented optic cups and discs in fundus images, thereby facilitating glaucoma characterization and assessing its severity. The model’s performance was tested on a combined dataset sourced from four publicly accessible repositories (RIM-ONE, DRISHTI-GS, DRIONS-DB, and HRF) and achieved impressive results with 98.58% accuracy, 98.17% specificity, 98.80% sensitivity, 98.86% precision, an F1-score of 98.83%, and an AUC of 0.98. Moreover, evaluations of individual datasets indicated remarkable accuracy rates: 98.67% for RIM-ONE, 97.71% for DRIONS-DB, 97.22% for HRF, and 97.50% for DRISHTI-GS. Notable advantages of this method include its high classification accuracy, enhanced sensitivity, and superior performance compared to existing deep learning techniques. However, it also has drawbacks such as the risk of overfitting related to model complexity and the need for optimization to enable real-time clinical application. In^40^, Sharma et al. revealed a customized CNN-based CAD model for the automatic detection of glaucoma. This model includes four trainable layers: three convolutional layers and a flattened layer for extracting deep features with minimal tunable parameters. Feature dimensionality is reduced through a combined PCA and LDA technique, while classification is processed using an Extreme Learning Machine (ELM) optimized with Modified Particle Swarm Optimization (MOD-PSO). The evaluation of the model on two standard datasets, G1020 and ORIGA, yielded accuracy rates of 97.80% and 98.46%, respectively. Strengths of this model include high classification accuracy, efficient feature extraction, reduced dimensionality, and improved classification performance. However, challenges include limited diversity in the datasets and the necessity for validation in real-world clinical settings.

In^41^, Sujithra et al. introduced a CAD system aimed at the early detection and diagnosis of glaucoma. This model employs preprocessing techniques to enhance the quality of fundus images, followed by blood vessel segmentation to extract critical structural characteristics. The resulting segmented data is then analyzed using Gray Wolf Optimization (GWO) in combination with ResNet50 through transfer learning for effective classification. Validation was carried out on various public benchmark datasets as well as a private dataset, demonstrating enhanced performance compared to existing methodologies and a boost in diagnostic accuracy. Nevertheless, limitations of the system include reliance on high-quality fundus images and possible biases in the dataset. In^42^, Rangaiah et al. proposed a glaucoma diagnosis framework combining median filtering for noise reduction, U-Net/U-Net+ for optic disc segmentation, Capsule Networks for feature extraction, and Extreme Learning Machines (ELM) for classification. The method achieved high accuracy on DRISHTI-GS (99%), DRIONS-DB (99.5%), and HRF (98.5%), with U-Net outperforming U-Net+ in segmentation. However, the approach is limited by its reliance on fundus images only, increased computational complexity, and evaluation restricted to a few public datasets, which may affect generalization. In^43^, Sivakumar et al. proposed a multi-modal glaucoma detection framework combining Vision Transformers (ViT), Object-Window-Location Vision Transformer (OWL-ViT), and Shifted Window Transformer (Swin) with Residual Networks (ResNet) for feature extraction. The Swin-ResNet hybrid achieved the best results, with 99.4% accuracy on 2874 cases. However, the approach is limited by its high computational cost and reliance on a single dataset, which may affect generalization.

### Discussion of limitations and contributions

Existing methods for automated glaucoma detection, despite significant advancements, face several limitations that hinder their widespread adoption and clinical effectiveness. One prominent challenge is data imbalance^44^, where many models fail to accurately detect early-stage glaucoma or rare conditions due to underrepresented cases in training datasets. Traditional data augmentation methods often struggle to replicate the variability observed in real-world clinical settings, leading to reduced model generalization^45^. Additionally, generalizability remains a critical issue, as models trained and validated on limited or homogeneous datasets frequently underperform when applied to diverse clinical populations. Another key limitation is computational complexity, which poses a significant challenge for real-time applications, particularly in resource-constrained environments, making many existing approaches impractical for widespread deployment.

To address these limitations, this paper proposes a novel, comprehensive framework designed to enhance accuracy, robustness, and practical usability. To mitigate data imbalance, the framework introduces a hybrid augmentation strategy that combines traditional techniques with dynamic Gaussian noise, effectively simulating real-world imaging variability and improving model generalization. Furthermore, to optimize scalability and computational efficiency, the framework employs a fine-tuned dual-stream CNN, integrated with the IMRFO algorithm for dynamic hyperparameter optimization. This approach ensures optimal model performance while minimizing computational overhead, making the framework more suitable for real-world clinical applications. By tackling data imbalance, enhancing generalizability, and improving computational efficiency, this framework represents a significant advancement toward more robust and clinically viable glaucoma detection systems.

## Preliminaries

This part outlines the essential principles of the techniques and approaches that form the basis of the proposed framework, with a special emphasis on the Manta Ray Foraging Optimization (MRFO) algorithm and the innovative Partial Centroid Opposition-Based Learning (PCOBL) strategy, which enhances MRFO’s performance. The discussion progresses through key concepts, starting from MRFO and culminating in PCOBL.

### Manta Ray Foraging Optimization (MRFO)

The MRFO algorithm draws inspiration from the foraging patterns of manta rays, which encompass three primary strategies: chain foraging, cyclone foraging, and somersault foraging. These strategies demonstrate the collaborative and efficient methods manta rays employ to seek food, positioning MRFO as a powerful optimization method for discovering global optima within intricate solution landscapes^46^.

**Chain Food Searching Approach:** In this approach, every manta ray updates its position by the best solution found to date and the position of the manta ray directly in front of it in the chain. Conversely, the first manta ray in the chain updates its position solely based on the best solution achieved. The update of position is defined by Eq. 1.

1 $${\mathrm{p}}_{\mathrm{k}}^{\mathrm{itr}+1 } =\left\{\begin{array}{c}{\mathrm{p}}_{\text{k }}^{\text{itr }}+\mathrm{rn} *\left({\mathrm{Gbest}}^{\mathrm{itr}} - {\mathrm{p}}_{\text{k }}^{\text{itr }} \right)+ 2 * \mathrm{rn} *\sqrt{|\mathrm{log}\left(r\right)| }*\left({\mathrm{Gbest}}^{\mathrm{itr}} - {\mathrm{p}}_{\text{k }}^{\text{itr }} \right) , \mathrm{k} = 1\\ {\text{ p}}_{\text{k }}^{\text{itr }}+\mathrm{rn} *\left({\mathrm{p}}_{\mathrm{k}-1 }^{\text{itr }} - {\mathrm{p}}_{\text{k }}^{\text{itr }} \right)+ 2 * \mathrm{rn} *\sqrt{|\mathrm{log}\left(r\right)| }*\left({\mathrm{Gbest}}^{\mathrm{itr}} - {\mathrm{p}}_{\text{k }}^{\text{itr }} \right), \mathrm{k} = 2, \dots , \mathrm{N}\end{array}\right.$$

where *rn* is a random number between 0 and 1, *N* is the total number of manta rays (i.e., population size), ${\mathrm{p}}_{\text{k }}^{\mathrm{itr}}$ is the position of *k *^*t*ℎ^ manta ray in the current iteration and ${\mathrm{p}}_{\mathrm{k}}^{\mathrm{itr}+1}$ define its new position in the coming iteration, and *Gbest* refer to the best global solution achieved**.**

**Cyclone Food Searching Approach**: In this strategy, manta rays traverse in a cyclic pattern, mimicking the swirling motion observed in cyclones. This approach enhances exploration by enabling manta rays to cover a broader area within the search space, as defined by Eq. 2.

2 $${\mathrm{p}}_{\mathrm{k}}^{\mathrm{itr}+1 }=\left\{\begin{array}{c}\mathrm{Gbest}+\mathrm{rn} *\left({\mathrm{Gbest}}^{\mathrm{itr}} - {\mathrm{p}}_{\text{k }}^{\text{itr }} \right)+ 2 * {e}^{\mathrm{rn}* \frac{\mathrm{MaxItr}-\mathrm{itr}+1}{\mathrm{MaxItr}}}* \mathrm{sin}\left(2 *\uppi *\text{ rn}\right)*\left({\mathrm{Gbest}}^{\mathrm{itr}} - {\mathrm{p}}_{\text{k }}^{\text{itr }} \right) , \mathrm{k} = 1\\ \mathrm{Gbest}+\mathrm{rn} *\left({\mathrm{p}}_{\mathrm{k}-1 }^{\text{itr }} - {\mathrm{p}}_{\text{k }}^{\text{itr }} \right)+ 2 * {e}^{\mathrm{rn}* \frac{\mathrm{MaxItr}-\mathrm{itr}+1}{\mathrm{MaxItr}}}* \sin\left(2 *\uppi *\text{ rn}\right)*\left({\mathrm{Gbest}}^{\mathrm{itr}} - {\mathrm{p}}_{\text{k }}^{\text{itr }} \right), \mathrm{k} = 2, .. , \mathrm{N}\end{array}\right.$$

where *MaxItr* represents the maximum number of iterations. The cyclic movement allows manta rays to revisit and explore areas of the search space that may have been previously overlooked, thereby improving the algorithm’s ability to avoid local optima. To further enhance the MRFO’s diversification capabilities, manta rays also perform random walks, updating their positions based on randomly chosen coordinates, as outlined in Eq. 3.

3 $${\mathrm{p}}_{\mathrm{k}}^{\mathrm{itr}+1 } =\left\{\begin{array}{c}{p}_{\mathrm{rn}} + \mathrm{rn} *\left({\mathrm{p}}_{\text{rn }}^{\text{itr }} - {p}_{\mathrm{rn}} \right)+ 2 * {e}^{\mathrm{rn}* \frac{\mathrm{MaxItr}-\mathrm{itr}+1}{\mathrm{MaxItr}}}* \mathrm{sin}\left(2 *\uppi *\text{ rn}\right)*\left({\mathrm{p}}_{\text{rn }}^{\text{itr }} - {\mathrm{p}}_{\text{k }}^{\text{itr }} \right), \mathrm{k} = 1\\ {p}_{\mathrm{rn}}+\mathrm{rn} *\left({\mathrm{p}}_{\mathrm{k}-1 }^{\text{itr }} - {\mathrm{p}}_{\text{k }}^{\text{itr }} \right)+ 2 * {e}^{\mathrm{rn}* \frac{\mathrm{MaxItr}-\mathrm{itr}+1}{\mathrm{MaxItr}}}* \sin\left(2 *\uppi *\text{ rn}\right)*\left({\mathrm{p}}_{\text{rn }}^{\text{itr }} - {\mathrm{p}}_{\text{k }}^{\text{itr }} \right), \mathrm{k} = 2, .. , \mathrm{N}\end{array}\right.$$

where *p*_*rn*_ represents a reference point in the search domain defined in Eq. 4.

4 $${p}_{\mathrm{rn}} =\text{LowerBound }+\text{rn }*\left(\text{UpperBound }-\text{ LowerBound }\right)$$

The limits of the search domain are established by the LowerBound and UpperBound parameters.

**Somersault Food Searching Approach:** In this strategy, every manta ray executes a somersault maneuver, significantly shifting its position toward the direction of the best solution discovered so far. This strategy enhances the algorithm’s exploitation capability^47^. The mathematical formulation of this approach is detailed in Eq. 5.

5 $${\mathrm{p}}_{\mathrm{k}}^{\mathrm{itr}+1 } ={\mathrm{p}}_{\text{rn }}^{\text{itr }}+\text{Somersault \,factor }* \left({\mathrm{rn}}_{1} *\text{ Gbest }- {\mathrm{rn}}_{2} * {\mathrm{p}}_{\text{rn }}^{\text{itr }}\right),\text{ i }= 1, \dots ,N$$

The somersault factor, often set to 2, controls the extent of the position adjustment.${\mathrm{rn}}_{1}$​ and ${\mathrm{rn}}_{2}$ are random numbers between 0 and 1. The pseudo-code is given in Algorithm 1.

**Algorithm 1:**
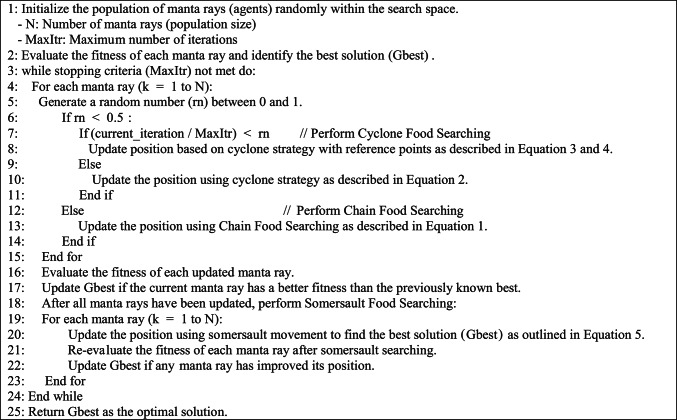
Pseudo-Code for MRFO algorithm

Despite its robustness, the MRFO algorithm’s performance can vary depending on hyperparameters and optimization landscapes. This has driven research efforts to incorporate advanced enhancement strategies, such as opposition-based learning, for improved convergence and accuracy.

### Opposition-based learning (OBL)

Opposition-Based Learning (OBL) introduces a simple yet powerful concept in optimization by generating opposite solutions to improve the exploration process. The opposite solution ${\check{\mathrm{x}}}$ for a point $\mathrm{x}$ within the interval $[a,b]$ is calculated as:

6 $${\check{\mathrm{x}}} = {\mathrm{a}} + {\mathrm{b}} - {\mathrm{x}}$$

By considering both $x$ and $x{\prime}$ during the optimization process, OBL effectively expands the search space, increasing the likelihood of identifying better solutions. In higher dimensions, OBL extends to calculate opposite points for all variables simultaneously^48^:

7 $${\check{\mathrm{x}}}_{{\text{i }}}^{ } = {\mathrm{a}}_{{\mathrm{i}}} + {\mathrm{b}}_{{\mathrm{i}}} - {\mathrm{x}}_{{\mathrm{i}}} \quad {\text{for i}} = 1,2, \ldots {\mathrm{D}}$$

OBL has shown significant promise in enhancing the performance of metaheuristic algorithms but is limited by its reliance on full opposites, which might not suit all problem landscapes. To address this, advanced strategies such as Centroid Opposition Based Learning (COBL) and Partial Opposition-Based Learning (POBL) were developed.

### Centroid opposition-based learning (COBL)

Centroid Opposition-Based Learning (COBL) enhances OBL by incorporating the concept of a centroid, which represents the average position of all solutions in the population^49^. The opposite solution in COBL is calculated relative to this centroid rather than the extremities of the search space. For a population $\mathrm{X}={\mathrm{x}}_{1},{\mathrm{x}}_{2},\dots {\mathrm{x}}_{\mathrm{N}}$ in a $D$-dimensional space, the centroid $M$ is defined as:

8 $${\mathrm{M}} = \frac{1}{N}\mathop \sum \limits_{i = 1}^{N} {\mathrm{x}}_{{\mathrm{i}}}$$

The opposite point for a solution ${\mathrm{x}}_{\mathrm{i}}$ is then computed as:

9 $${\check{\mathrm{x}}}_{{\text{i }}}^{ } = 2{\mathrm{M}} - {\mathrm{x}}_{{\mathrm{i}}}$$

COBL improves search dynamics by generating opposites that are more relevant to the population’s current distribution, enhancing convergence while maintaining diversity.

### Partial opposition-based learning (POBL)

Partial Opposition-Based Learning (POBL) extends the concept of traditional Opposition-Based Learning (OBL) by introducing the notion of partial opposites^50^. Rather than inverting all dimensions of a candidate solution as in standard OBL, POBL selectively inverts only a subset of dimensions to generate partial opposite points. This strategy increases diversity while avoiding excessive disruption of promising solutions. Si and Dutta^51^ applied POBL within the Particle Swarm Optimization (PSO) framework to optimize Artificial Neural Networks (ANN) for medical data classification. Their results showed that POBL significantly enhanced the exploration capability of PSO, leading to superior classification performance compared to conventional approaches. In a multi-dimensional search space, OBL typically computes a full opposite point by inverting each component of a solution vector across all dimensions. In contrast, POBL generates partial opposite points, where only certain dimensions are flipped, while others are left unchanged. This allows for finer control over the search behavior. Let ${\mathrm{P}\check{\mathrm{X}}}$ denote the set of partial opposite points corresponding to a point $X$ in a D-dimensional space. The calculation of these partial opposite points can be expressed as follows:

10 $${\mathrm{P}\check{\mathrm{X}}}^{1} = \left[ {\begin{array}{*{20}c} {{\mathrm{P}\check{\mathrm{X}}}_{1}^{1} } \\ {{\mathrm{P}\check{\mathrm{X}}}_{2}^{1} } \\ \ldots \\ {{\mathrm{P}\check{\mathrm{X}}}_{D}^{1} } \\ \end{array} } \right] = { }\left[ {\begin{array}{*{20}c} {{\mathrm{x}}_{{1, }} {\check{\mathrm{x}}}_{{2{ },{ }}} {\check{\mathrm{x}}}_{{3{ }}} } & \cdots & {{\check{\mathrm{x}}}_{D} } \\ {{\check{\mathrm{x}}}_{{1{ },{ }}} {\mathrm{x}}_{{{ }2{ },{ }}} {\check{\mathrm{x}}}_{{3{ }}} } & \cdots & {{\check{\mathrm{x}}}_{D} } \\ \vdots & \ddots & \vdots \\ {{\check{\mathrm{x}}}_{{1{ },{ }}} {\check{\mathrm{x}}}_{{2{ },{ }}} {\check{\mathrm{x}}}_{{3{ }}} } & \cdots & {x_{D} } \\ \end{array} } \right]$$

The superscript 1 in ${\mathrm{P}\check{\mathrm{X}}}^{1}$ denotes the level of partial opposition. The depicted partial opposite points hold an order or degree of one because each point contains only one original number in one dimension. Therefore, a point $\mathrm{X}$ in $\mathrm{D}$-dimensional space has D number of partial opposite points. Let $\mathrm{X}=({ \mathrm{x}}_{1 , }{\mathrm{x}}_{2 , }{ \mathrm{x}}_{3 })$ be a 3-dimensional point and ${\check{\mathrm{X}}}=({\check{\mathrm{x}}}_{1 , }{\check{\mathrm{x}}}_{2 , }{\check{\mathrm{x}}}_{3 })$ be its opposite point. The partial opposite points are ${\mathrm{P}\check{\mathrm{X}}}_{1}^{1}=({\mathrm{x}}_{ 1 , }{\check{\mathrm{x}}}_{2 , }{\check{\mathrm{x}}}_{3 })$ , ${\mathrm{P}\check{\mathrm{X}}}_{2}^{1}=\left({\check{\mathrm{x}}}_{1, }{\mathrm{x}}_{ 2, }{\check{\mathrm{x}}}_{3 }\right)$ and ${\mathrm{P}\check{\mathrm{X}}}_{3}^{1}=({\check{\mathrm{x}}}_{1 , }{\check{\mathrm{x}}}_{2 , }{ \text{x }}_{3})$ . By introducing controlled variations through partial opposites, POBL balances exploration and exploitation more effectively, particularly in high-dimensional optimization problems.

### Partial centroid opposition-based learning (PCOBL)

Partial Centroid Opposition-Based Learning (PCOBL) integrates the foundational concepts of COBL and POBL, leveraging the advantages of both methods to improve the exploration and exploitation capabilities of metaheuristic algorithms. In PCOBL, the overall structure is similar to that of POBL however, it utilizes opposite values derived from the COBL scheme rather than the traditional OBL scheme. As with other OBL variants, PCOBL aims to increase the likelihood of discovering higher-quality solutions by generating a set of partial centroid opposite solutions. The process begins by calculating the centroid of the population, following the method used in COBL. A complete centroid opposite solution is then constructed. From this, multiple partial centroid opposite solutions are generated by randomly selecting a subset of dimensions to retain from the original solution, while the remaining dimensions are replaced by their centroid opposite counterparts^52^. This mechanism results in a spectrum of solutions ranging from highly exploratory (low similarity to the original) to more exploitative (high similarity to the original). Such diversity enables the search process to adaptively shift between exploration and exploitation, thus improving the overall performance of the underlying metaheuristic algorithm. The pseudo code for the PCOBL mechanism is presented in Algorithm 2.

**Algorithm 2:**
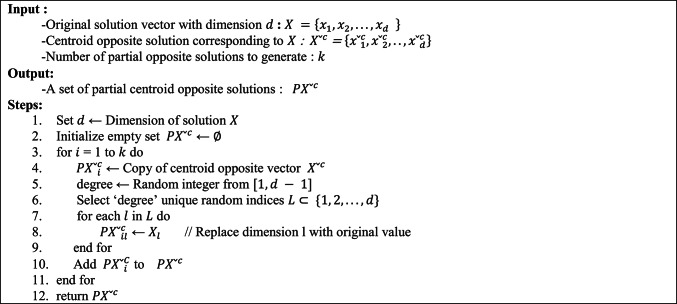
Pseudo Code for PCOBL

By integrating PCOBL into the generation jumping phase of metaheuristic algorithm, the algorithm enhances the search efficiency and solution quality by leveraging both global and local search capabilities inherent in the partial opposition concept.

## The proposed model

**Fig. 2:**
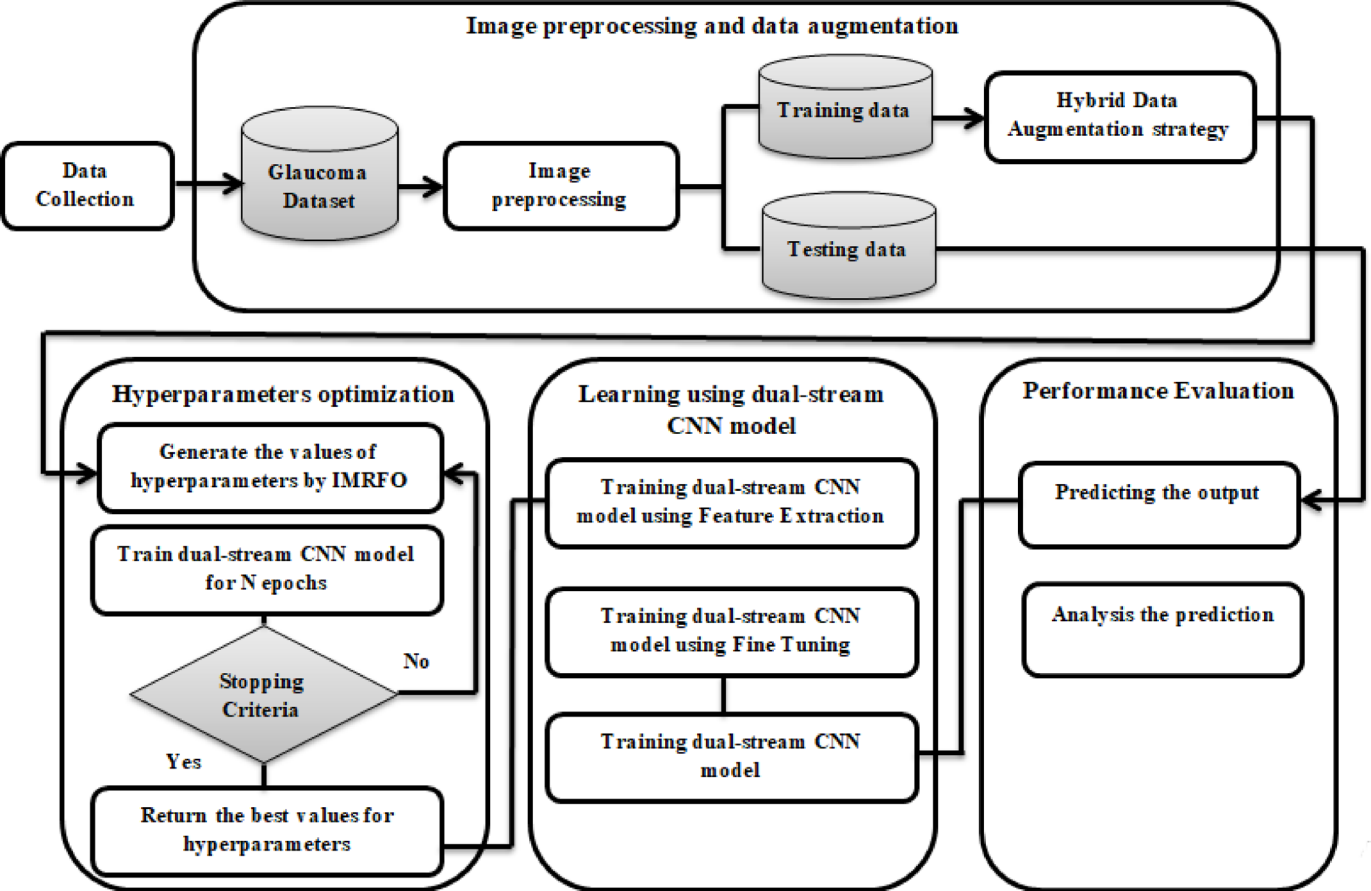
The main steps for the proposed model.

The proposed framework follows a sequential process, starting with data preprocessing to standardize input images and adapt them to real-world variability. It introduces a hybrid augmentation strategy that combines traditional techniques, such as rotations, flips, and shifts, with dynamic Gaussian noise injection. This approach effectively simulates real-world imaging conditions and enhances the model’s robustness (Fig. 2).

**Algorithm 3:**
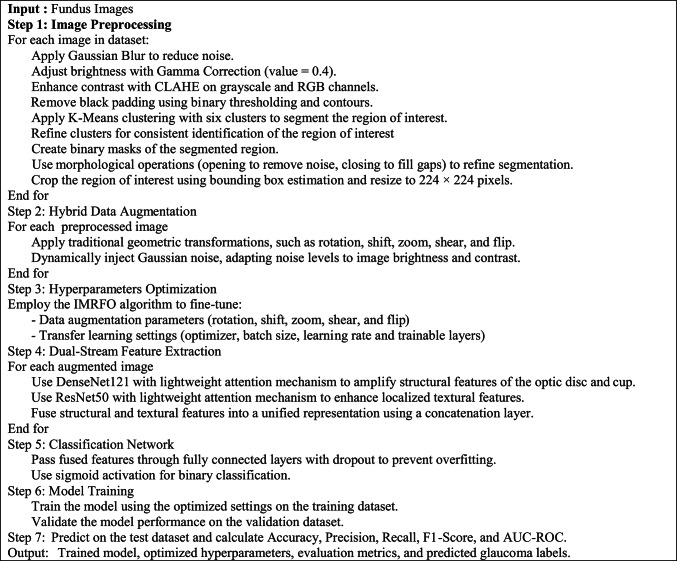
Pseudo-Code for Proposed Framework for Glaucoma Detection

### Data collection

The study leverages four publicly accessible datasets: ACRIMA^53^, ORIGA^54^, RIM-ONE DL^55^, and DRISHTI-GS^56^, each contributing valuable data for glaucoma detection research. The ACRIMA dataset consists of 705 fundus images, including 396 from glaucomatous cases and 309 from normal, healthy individuals. These retinal images centered on the optic disc (OD), were collected from both the left and right eyes at a resolution of 2048 × 1536 pixels and were reviewed by two ophthalmologists at the Mediterranean Ophthalmological Foundation (FOM). The ORIGA dataset offers 650 annotated images, particularly valuable for detecting peripapillary atrophy (PPA) and identifying disc border blood vessel junctions. The RIM-ONE DL dataset provides 485 high-resolution images, segmented to highlight the optic nerve head, including 172 glaucomatous and 313 normal cases. These images, captured using a Nidek AFC-210 fundus camera, were segmented by experts. Lastly, the DRISHTI-GS dataset includes 101 retinal images, comprising 31 healthy and 70 glaucomatous cases, with detailed annotations of the optic disc in high-resolution PNG format. Collectively, these datasets are publicly accessible and serve as a crucial resource for advancing glaucoma detection methods.

### Image preprocessing and data augmentation

**Fig. 3:**
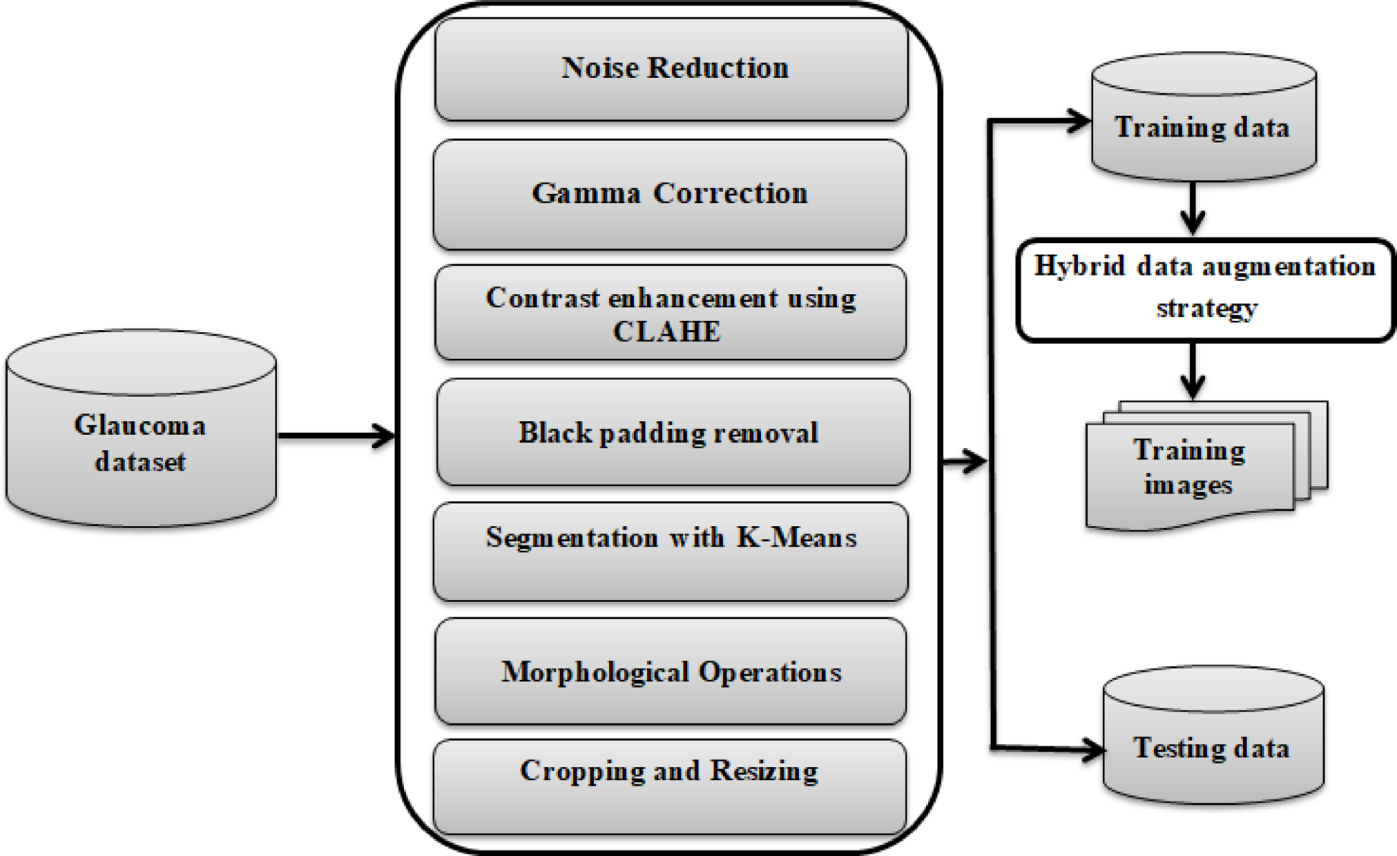
The primary stages of the preprocessing workflow.

In the proposed framework for glaucoma detection, image preprocessing and data augmentation serves as a crucial step in preparing retinal fundus images for precise classification. The preprocessing pipeline incorporates a series of essential steps, employing specialized algorithms to enhance image quality and isolate significant features critical for accurate analysis. Figure 3 depicts the primary stages of the preprocessing workflow.

**Fig. 4:**
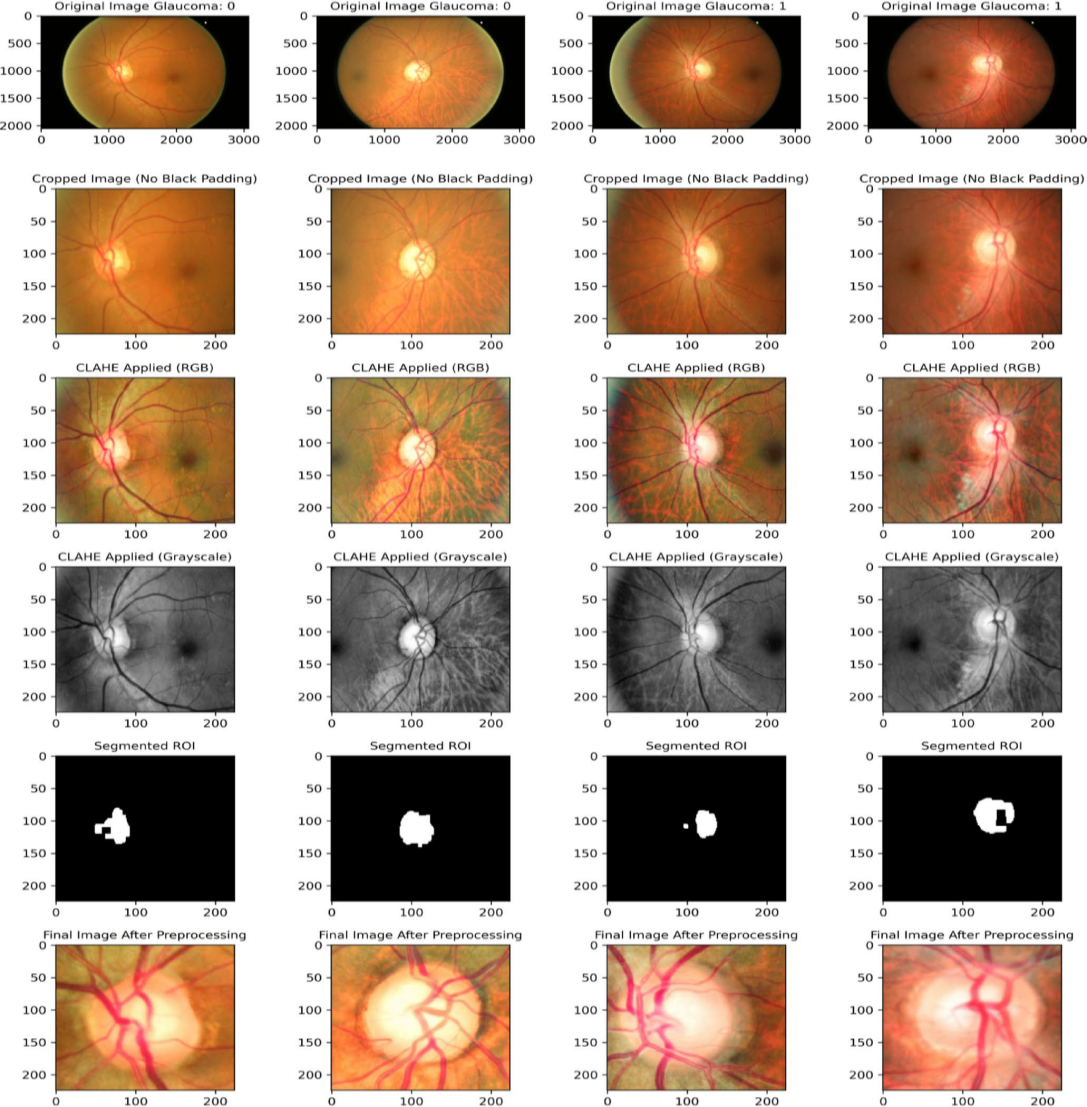
Visualization of the preprocessing pipeline steps on examples from the ORIGA dataset.

The preprocessing workflow in the proposed framework employs a comprehensive series of steps to prepare retinal fundus images for glaucoma detection, ensuring enhanced image quality, precise segmentation, and optimal feature extraction. The process begins with noise reduction using Gaussian Blur, which smoothens the images while retaining critical structural details. Brightness normalization is then achieved through Gamma Correction (γ = 0.4), standardizing global illumination across the dataset. To further enhance local contrast while avoiding noise amplification, Contrast Limited Adaptive Histogram Equalization (CLAHE) is applied to both grayscale and RGB channels. This complementary combination of Gamma Correction and CLAHE ensures balanced brightness and improved visibility of clinically relevant structures. To focus on informative regions and remove non-informative artifacts, images undergo black padding removal using binary thresholding and contour-based bounding box extraction, retaining only the retinal structures. Additionally, the pipeline incorporates a robust segmentation process to isolate the optic disc and cup, which are key regions for glaucoma analysis. Segmentation begins with K-Means clustering^57^, grouping image pixels into six distinct clusters based on intensity. A cluster refinement step standardizes the cluster order to ensure consistent identification of the optic disc and cup across images. Binary masks are then generated to highlight the target regions, followed by morphological operations (opening to remove noise and closing to fill gaps), producing a refined segmentation of the optic disc and cup. The localized regions are finally cropped using bounding box estimations and resized to 224×224 pixels, maintaining uniformity across the dataset. Figure 4 illustrates the preprocessing pipeline applied to representative examples from the ORIGA dataset.

**Fig. 5:**
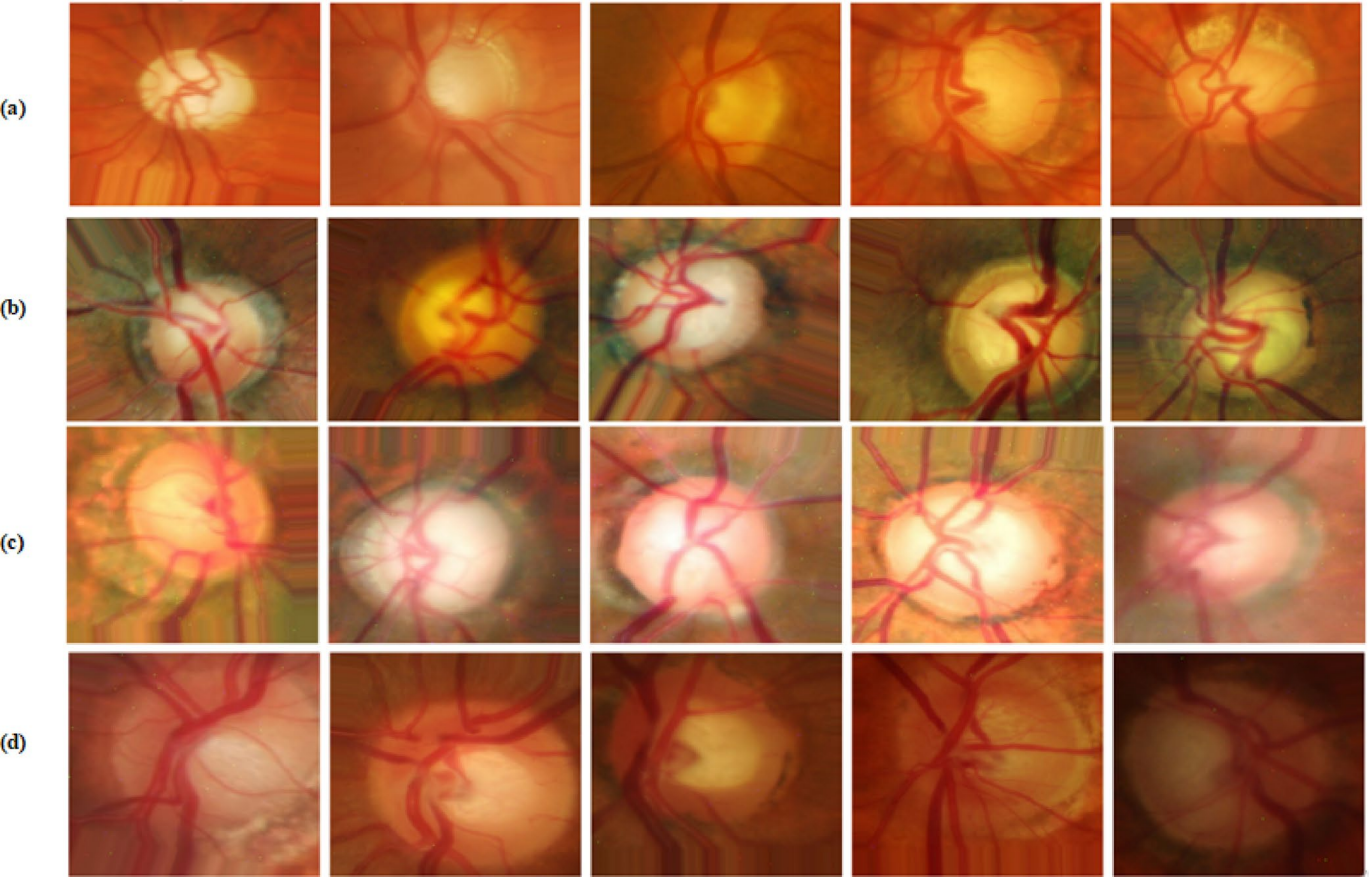
Examples of newly augmented images generated by the proposed augmentation pipeline across four datasets: (**a**) ACRIMA, (**b**) Drishti-Gs, (**c**) ORIGA, (**d**) RIM-ONE-DL.

A Hybrid Data Augmentation Strategy is proposed, combining conventional geometric transformations with an adaptive noise injection mechanism. The geometric augmentation includes standard operations such as rotation, zooming, shifting, flipping, and shearing to enhance spatial diversity and reduce overfitting. In addition to these transformations, a novel Adaptive Gaussian Noise Injection technique is introduced to simulate realistic noise patterns commonly observed in clinical imaging environments. This dynamic noise mechanism adaptively adjusts the intensity of Gaussian noise added to each image based on two fundamental properties: brightness and contrast. The brightness is estimated as the mean pixel intensity, while the contrast is computed as the standard deviation of pixel intensities. The intuition behind this approach is that brighter images, often captured under stronger illumination, are typically more susceptible to sensor noise and thus require higher noise levels, whereas high-contrast images, which tend to contain more critical structural information, are perturbed less to preserve essential features. The noise intensity (σ) is computed dynamically for each image using the following formulation:

11 $$\upsigma =\mathrm{k}*\left(1+\frac{\mathrm{Brightness}}{255}\right)*\left(1-\frac{\mathrm{Contrast}}{255}\right)$$

where $\mathrm{k}$ is a scaling factor (e.g., 0.001 × 255) used to control the base noise level. This formulation ensures that brighter and low-contrast images receive higher noise, while darker or high-contrast images receive less perturbation, striking a balance between realism and feature preservation. By integrating this adaptive noise component into the augmentation pipeline, the method enhances the model’s robustness and generalization across varied and imperfect imaging conditions, mimicking the challenges encountered in real-world clinical data acquisition. The detailed steps of the proposed data augmentation process are outlined in Algorithm 4, which systematically describes how the traditional transformations and adaptive noise are applied to balance the dataset and enhance variability. Representative examples of augmented images generated by this strategy are illustrated in Fig. 5.

**Algorithm 4:**
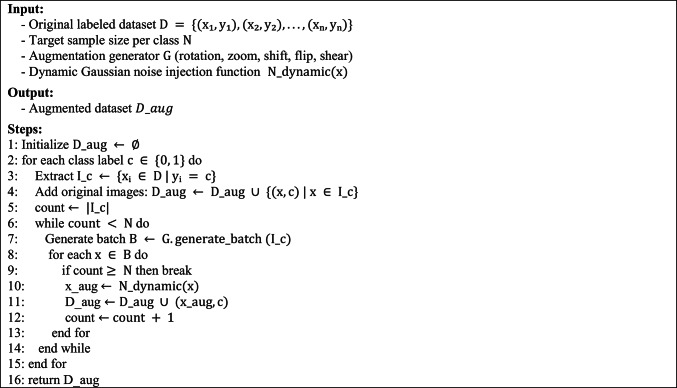
Hybrid Data Augmentation Strategy

This rigorous preprocessing pipeline ensures that the resulting images exhibit enhanced quality, precise segmentation, and uniformity, laying a robust foundation for the dual-stream CNN architecture to perform accurate and reliable glaucoma detection.

### Hyperparameters optimization using IMRFO

To achieve optimal performance, the proposed framework integrates an Improved Manta Ray Foraging Optimization (IMRFO) algorithm, which combines Manta Ray Foraging Optimization (MRFO) with Partial Centroid Opposition-Based Learning (PCOBL). While the standard MRFO algorithm simulates three natural foraging behaviors of manta rays chain foraging, cyclone foraging, and somersault foraging its performance may deteriorate when the population lacks diversity or becomes trapped in local optima. To address this issue, PCOBL is integrated as an auxiliary search mechanism to periodically introduce diverse candidate solutions. This hybrid strategy enables IMRFO to periodically inject new and diverse solutions into the population, enhancing its ability to escape local optima and accelerating convergence toward global optima. Hyperparameter selection plays a crucial role in deep learning models, impacting both training stability and final performance. The optimization process in this framework is designed to search for the most effective hyperparameter configurations while balancing exploration (diversity in search space) and exploitation (refinement of promising solutions) to avoid suboptimal local minima. The key hyperparameters optimized by IMRFO include:

- **Data Augmentation Parameters:** Rotation angle, shift range (horizontal/vertical), zoom factor, flipping (horizontal/vertical), and shear intensity.
- **Transfer Learning Configurations:** Choice of optimizer (Adam, SGD, and RMSprop), learning rate, batch size, and the proportion of trainable layers in DenseNet121 and ResNet50.

Unlike conventional methods such as grid search or random search, which are computationally expensive, IMRFO intelligently adjusts hyperparameters in real time based on feedback from validation performance, achieving optimal trade-offs between model complexity and accuracy.

In the IMRFO framework, the integration of PCOBL takes place during two key stages of the optimization process. During the initial phase, known as opposition-based initialization, the original population is enriched using the COBL mechanism to produce centroid-opposite solutions. These solutions are then combined with the original individuals, and the best-performing candidates are selected to form a more diverse and promising initial population, thereby increasing the chances of starting the search closer to optimal regions in the solution space. As the optimization progresses, the generation jumping mechanism is applied based on a predefined generation-jumping probability ​${\mathrm{P}}_{\mathrm{gj}}$ . At this stage, each individual in the population undergoes partial centroid opposition transformation, which involves generating several PCOBL-based solutions by replacing a subset of the individual’s dimensions with their centroid-opposite values while keeping the remaining dimensions unchanged. The population is then updated by selecting the best individuals from the combined pool of original, centroid-opposite, and partial centroid-opposite solutions. Algorithm 5 is a pseudo code that exemplifies how to use PCOBL in a population-based MRFO algorithm.

**Algorithm 5:**
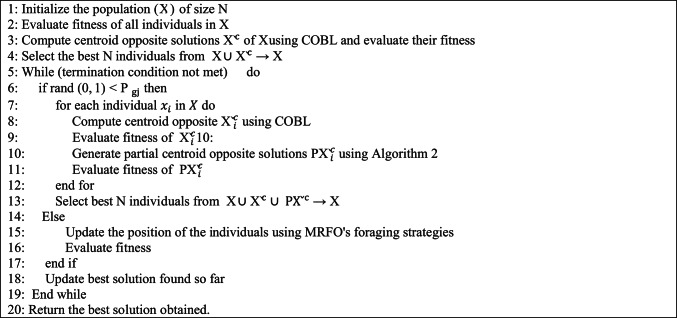
Pseudo Code for IMRFO

In this algorithm, the initial step involves initializing the population X. Following that, in step 2, the fitness of the search agents is evaluated, and in step 3, the centroid opposite solutions ${\check{\mathrm{X}}}^{\mathrm{c}}$ of $\mathrm{X}$ are generated using COBL schemes, along with the calculation of their fitness values. Step 4 involves selecting the best *N* solutions from the combined set $\{\mathrm{X},{\check{\mathrm{X}}}^{\mathrm{c}}\}$. Steps 6–19 are repeated until the termination criteria are satisfied. Steps 8–13 consist of executing PCOBL if the condition in step 6 holds true. In step 8, opposite solutions are generated using COBL, and in step 9, their fitness values are determined. Step 10 involves calculating the number of opposite solutions ${\mathrm{P}\check{\mathrm{X}}}^{\mathrm{c}}$ through PCOBL, followed by the calculation of their fitness values in step 11. In step 13, the best $N$ solutions are chosen from the set $\{\text{X }, {\check{\mathrm{X}}}^{\mathrm{c}}, {\mathrm{P}\check{\mathrm{X}}}^{\mathrm{c}}\}$. If the condition in step 6 is not met, steps 15–18, which include the basic operations of the MRFO, are carried out. Integrating PCOBL with MRFO significantly enhances hyperparameter optimization, leading to:

- Faster Convergence**:** PCOBL accelerates the search process by introducing controlled diversity, reducing the number of iterations needed for optimization.
- Improved Accuracy and Robustness**:** Fine-tuning augmentation and regularization parameters ensures generalization across unseen data, improving model reliability.
- Computational Efficiency: The adaptive nature of IMRFO eliminates the need for brute-force hyperparameter tuning, reducing computational overhead without sacrificing performance.

By leveraging IMRFO, the proposed framework ensures optimal hyperparameter selection, enhancing the efficiency of the dual-stream CNN architecture for real-world clinical deployment.

### The learning using dual-stream CNN with lightweight channel-wise attention

The proposed framework utilizes an innovative dual-stream CNN architecture to effectively gather both structural and texture-related features from retinal fundus images, capitalizing on the advantages of two leading networks: DenseNet121 and ResNet50. The proposed dual-stream CNN is architecturally designed to explicitly disentangle structural and texture-related representations from retinal fundus images. Unlike conventional dual-stream approaches that employ parallel networks for generic feature extraction, each stream in the dual-stream CNN is purposefully assigned a distinct functional role The Structural Stream, based on DenseNet121, is particularly adept at extracting large-scale features like the optic disc and cup. DenseNet121, a densely connected convolutional neural network, enables efficient feature reuse by linking each layer to every other layer in a feed-forward manner^58^. This architecture reduces feature redundancy, improves gradient flow, and boosts learning efficiency, making it highly effective for detecting large-scale patterns associated with the optic nerve. In contrast, the Texture Stream, which is based on ResNet50, emphasizes the capture of smaller micro-textural details. ResNet50 utilizes deep residual learning through skip connections, which effectively tackle the vanishing gradient issue and permit efficient training of deeper networks^59^. Its ability to represent subtle variations and localized patterns is essential for recognizing micro-textures and differences that suggest glaucomatous changes. By explicitly separating and fusing structural and textural features, the proposed dual-stream design yields a complementary and enriched representation, which constitutes the core novelty of the dual-stream CNN and provides a robust basis for subsequent enhancements.

To enhance feature discrimination, both streams incorporate a lightweight channel-wise attention mechanism. Unlike conventional attention modules (e.g., Squeeze-and-Excitation or CBAM), which add significant architectural overhead^60^, the proposed attention design is deliberately simplified for efficiency. Specifically, given the extracted feature vectors from DenseNet121 ${(F}_{s})$ and ResNet50 $({F}_{t})$, channel attention weights are computed using a sigmoid-activated dense layer as follows:

12 $${A}_{s} = \sigma \left({W}_{s} \cdot {F}_{s}\right) , {A}_{t} = \sigma \left({W}_{t} \cdot {F}_{t}\right)$$

where ${W}_{s}$ and ${W}_{t}$ are trainable weight parameters. These attention weights are applied via element-wise multiplication to modulate the original features:

13 $${F{\prime}}_{s} ={A}_{s} \cdot {F}_{s} , {F{\prime}}_{t} ={A}_{t} \cdot {F}_{t}$$

The re-weighted structural (${F{\prime}}_{s}$) and texture $\left({{F}{\prime}}_{t}\right)$ features are subsequently fused to form a high-dimensional representation:

14 $${F}_{fusion} =concatenate \left({F{\prime}}_{s} , {F{\prime}}_{t}\right)$$

**Fig. 6:**
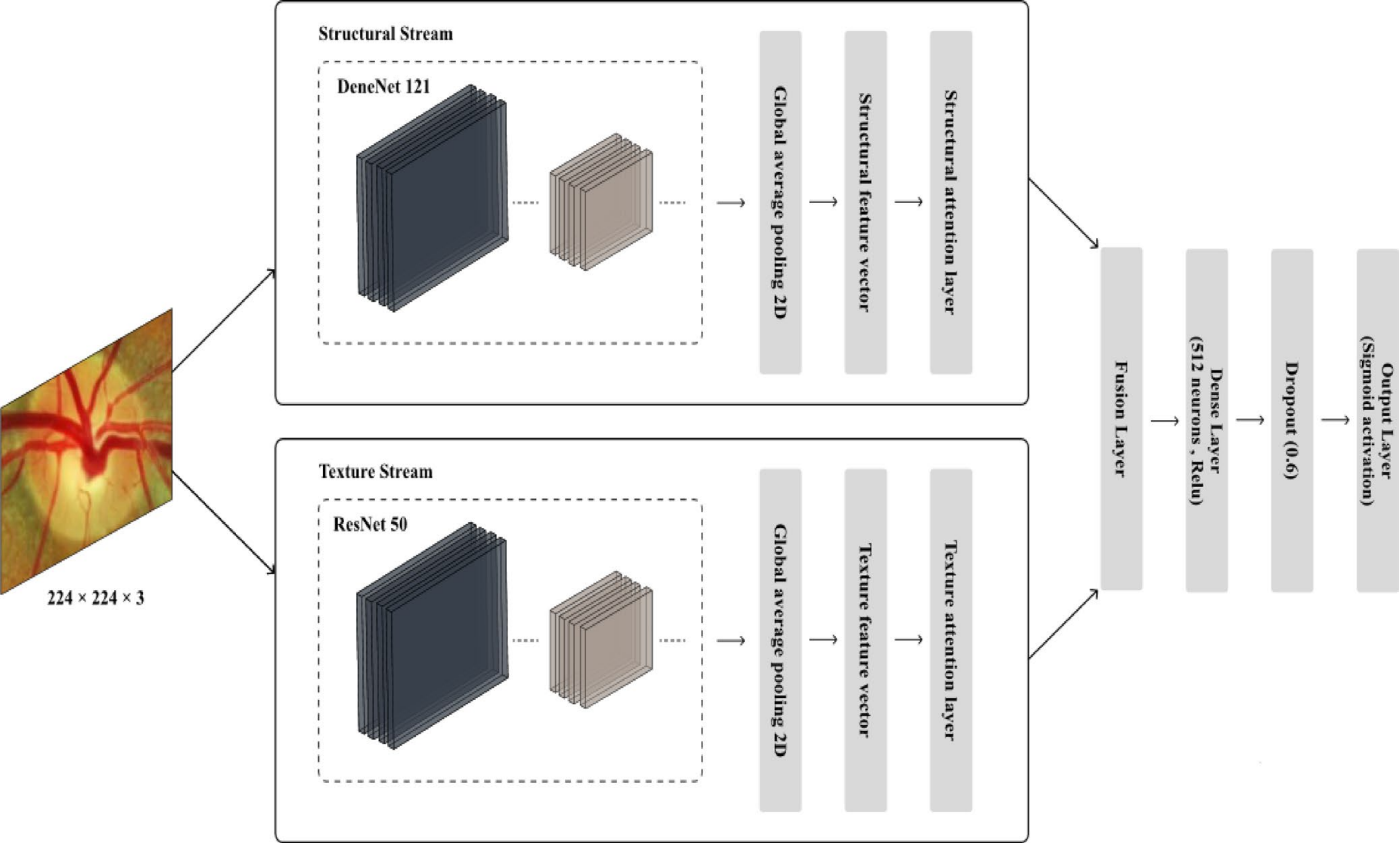
The architecture of dual-stream CNN with lightweight channel-wise attention.

This lightweight attention-driven fusion mechanism enables the network to emphasize clinically significant channels, such as those capturing the optic disc and fine retinal textures, while suppressing redundant or less informative ones. In doing so, the framework balances computational efficiency with interpretability, ensuring that the model remains effective without introducing unnecessary complexity. The outputs of the Structural and Texture Streams are then fused into a unified high-dimensional representation, leveraging complementary features from both pathways, as illustrated in Fig. 6. Subsequently, the fused representation is passed through fully connected layers with dropout regularization to reduce overfitting and enhance generalization. Finally, a sigmoid-activated output layer performs robust binary classification of glaucoma status. By integrating dual-stream feature extraction with a simplified yet effective channel-attention module, the proposed model design not only achieves superior predictive performance compared to previous approaches but also maintains a lightweight architecture. This demonstrates that task-specific lightweight attention can outperform heavier conventional modules, making the framework a promising contribution for clinical retinal image analysis and real-world deployment.

### Performance evaluation

Performance evaluation is a critical step within the proposed framework, aimed at assessing the effectiveness and robustness of the model. This phase takes the output from the dual-stream CNN with lightweight channel-wise attention and systematically evaluates it to ensure the model’s reliability across diverse scenarios. The evaluation process starts with dividing the dataset into training, validation, and test sets through stratified splitting to ensure class distribution is consistent across each subset. The training set is used for developing the model, the validation set serves to adjust hyperparameters and mitigate overfitting, and the test set, which the model has not been exposed to during training, is utilized to assess the model’s ability to generalize.

The model’s effectiveness is assessed employing common metrics including accuracy, precision, recall (sensitivity), AUC (Area under the ROC Curve), and loss. These metrics are crucial for assessing the model’s capability to differentiate between glaucomatous and non-glaucomatous cases. The mathematical expressions for each metric are included to provide clarity in their calculation. In this context, True Positives (TP) and True Negatives (TN) represent cases that have been accurately identified, while False Positives (FP) and False Negatives (FN) signify instances that have been incorrectly categorized^61^. These fundamental terms are essential for computing the performance metrics, as outlined in Table 1.

**Table 1:** Summary of basic terminologies for performance metrics.

	Predicted class			
Actual class		Non-glaucoma	Glaucoma	Total
	Non-Glaucoma	TP	FN	P
	Glaucoma	FP	TN	N
	Total	TP + FP	FN + TN	P + N

The performance metrics are calculated as follows:

**Accuracy:** Indicates the percentage of correctly classified samples.

15 $$\mathrm{Accuracy}=\frac{\mathrm{TP}+\mathrm{TN}}{\mathrm{P}+\mathrm{N}}$$

**Precision:** Measures the positive prediction accuracy.

16 $$\mathrm{Precision}=\mathrm{TP}/(\mathrm{TP}+\mathrm{FP})$$

**Recall (Sensitivity):** Measures the completeness of positive case identification.

17 $$\mathrm{Recall}=\mathrm{TP}/(\mathrm{TP}+\mathrm{FN})=\mathrm{TP}/\mathrm{P}$$

**AUC:** Assesses the model’s capability to differentiate between categories, with greater values signifying improved performance^62^. This measure is calculated based on ranking the predicted probabilities:

18 $$\mathrm{AUC}=\frac{\sum_{i=1}^{{n}_{positive}}{\mathrm{Rank}}_{i}-\frac{\begin{array}{c} \\ {n}_{positive}({n}_{positive}+1)\end{array}}{2}}{{n}_{positive}+ {n}_{negative}}$$

where ${n}_{positive}$ refers to the number of positive samples (e.g., glaucomatous cases), ${n}_{negative}$ refers the number of negative samples (e.g., non-glaucomatous cases), and ${\mathrm{Rank}}_{i}$ refers to the rank of the predicted probability for the positive samples when all probabilities are ranked together.

**Loss:** Reflects the error in prediction, calculated using binary cross-entropy loss for classification tasks.

Through these comprehensive evaluation techniques, the proposed framework’s ability to accurately and reliably detect glaucoma is rigorously validated, ensuring its suitability for real-world clinical application.

## Experimental results and performance analysis

### Experimental setup

To assess the effectiveness of the proposed model, comprehensive experiments were carried out on the gathered medical image datasets. The dataset was split into training (70%), validation (15%), and testing (15%) sets to guarantee a thorough evaluation of the model as described in Table 2. All experiments were executed on a Dell Latitude E6440 laptop with an Intel Core i7-4300 M CPU running at 2.60 GHz and 8 GB of RAM.

**Table 2:** Number of images used for training, validation, and testing for each dataset.

Dataset	Train (70%)	Val (15%)	Test (15%)	Total
ACRIMA	494	105	106	705
DRISHTI-GS	71	15	15	101
ORIGA	455	98	97	650
RIM-ONE DL	340	72	73	485

### Parameter settings

In this section, the experimental parameter settings utilized for the IMRFO algorithm are presented. These settings were carefully selected to optimize the model’s performance during the training and evaluation phases. A summary of the key parameters, their defined ranges, and corresponding descriptions used throughout the experiments is provided in Table 3.

**Table 3:** Summary of the experimental parameter settings for the IMRFO algorithm.

Parameter	Range/Values	Description
Maximum Iterations	50	Maximum number of iterations for optimization
Population Size	20	Number of individuals in the population
Rotation	[0° to 30]	Rotational augmentation angles
Width Shift	[0 to 0.2]	Horizontal translation of images
Height Shift	[0 to 0.2]	Vertical translation of images
Zoom	[0 to 0.2]	Zoom augmentation range
Shear	[0 to 0.2]	Shearing transformation range
Horizontal Flip	[True / False]	Horizontal flipping of images
Vertical Flip	[True / False]	Vertical flipping of images
Optimizer	[Adam, SGD, RMSprop]	Optimization algorithms
Batch Size	[16,32,64]	Number of samples per gradient update
TL Learn Ratio	[0 to 30]	Transfer learning rate ratio
Learning rate	[0.0001 to 0.01]	Learning rate for training

These settings facilitated comprehensive experimentation, allowing for the identification of optimal hyperparameter configurations that enhance the proposed model performance.

### Results and comparative analysis

In this section, the efficacy of the proposed framework is comprehensively assessed through four core performance experiments, each targeting distinct aspects of the proposed model. These experiments are conducted on four benchmark datasets: ACRIMA, Drishti-Gs, ORIGA, and RIM-ONE-DL, ensuring robust validation across diverse clinical scenarios. The first experiment analyzes the performance of various CNN architectures and highlights the superiority of the proposed dual-stream model in capturing both structural and textural features. The second experiment evaluates the impact of the hybrid data augmentation (HDA) strategy in enhancing generalization. The third experiment focuses on integrating a lightweight channel-wise attention mechanism to improve feature refinement. The fourth experiment examines the effectiveness of the IMRFO algorithm in hyperparameter tuning, showcasing its role in optimizing model performance.

To further isolate and quantify the contribution of each major component, an ablation study is conducted. This study systematically analyzes the incremental effects of HDA, the attention mechanism, and IMRFO optimization on model performance across the four benchmark datasets. Beyond performance evaluation, statistical validation is performed using paired t-tests to verify that the observed performance improvements introduced by each component are statistically significant. Finally, a comparative study with state-of-the-art methods is presented to demonstrate the robustness, competitiveness, and clinical reliability of the proposed framework. Collectively, these evaluations provide a comprehensive and transparent assessment of both the performance gains and the individual contributions of the proposed components.

#### First experiment: for the baseline model performance and comparison with dual-stream model

In this experiment, the performance of various CNN models MobileNet**,** DenseNet-201**,** ResNet-50, and EfficientNet-B0 was evaluated to establish a baseline for comparison. The primary objective was to identify the most effective architectures for glaucoma detection, which would later be integrated into the proposed dual-stream CNN. The evaluation was conducted on four benchmark datasets: ACRIMA, Drishti-Gs, ORIGA, and RIM-ONE-DL, ensuring comprehensive assessment across diverse clinical scenarios. All datasets underwent standard preprocessing, including noise reduction, brightness adjustment, contrast enhancement, black padding removal, and optic disc and cup segmentation. The models were trained utilizing the Adam optimizer, set with a learning rate of 0.01, a batch size of 32, and for a total of 40 epochs to guarantee uniform learning throughout all models. The evaluation of performance was conducted using essential metrics such as accuracy, precision, recall, AUC, and loss. The results are presented in Tables 4, 5, 6, and 7, corresponding to the ACRIMA, Drishti-Gs, ORIGA, and RIM-ONE-DL datasets, respectively. These tables summarize model performance across datasets, highlighting the dual-stream CNN’s superiority over single-stream architectures.

**Table 4:** Performance of different CNN models on the ACRIMA Dataset.

CNN Model	Accuracy (%)	Precision (%)	Recall (%)	AUC (%)	Loss
EfficientNet-B0	88.1	85.5	82.2	85.7	0.41
MobileNet	86.6	83.9	80.7	82.4	0.48
ResNet-50	93.6	91.6	89.4	91.8	0.39
DenseNet-201	92.9	90.4	88.7	90.5	0.42
Dual-Stream (DenseNet-201 + ResNet-50)	95.9	92.7	91.2	93.8	0.36

**Table 5:** Performance of different CNN models on the Drishti-Gs Dataset.

CNN Model	Accuracy (%)	Precision (%)	Recall (%)	AUC (%)	Loss
EfficientNet-B0	86.1	83.4	82.7	84.7	0.46
MobileNet	84.6	81.8	80.4	82.0	0.45
ResNet-50	89.9	87.3	85.9	87.8	0.42
DenseNet-201	93.1	90.9	89.7	92.5	0.37
Dual-Stream (DenseNet-201 + ResNet-50)	96.7	94.7	93.2	95.9	0.32

**Table 6:** Performance of different CNN models on the ORIGA Dataset.

CNN Model	Accuracy (%)	Precision (%)	Recall (%)	AUC (%)	Loss
EfficientNet-B0	83.1	80.5	79.2	81.6	0.48
MobileNet	78.6	76.7	75.4	77.1	0.52
ResNet-50	87.9	84.8	83.5	85.6	0.47
DenseNet-201	88.4	86.2	84.9	86.8	0.45
Dual-Stream (DenseNet-201 + ResNet-50)	93.8	91.7	90.5	92.8	0.38

**Table 7:** Performance of different CNN models on the RIM-ONE-DL Dataset.

CNN Model	Accuracy (%)	Precision (%)	Recall (%)	AUC (%)	Loss
EfficientNet-B0	84.1	82.6	81.3	83.7	0.47
MobileNet	86.6	83.4	82.9	83.8	0.43
ResNet-50	87.9	84.8	83.8	86.0	0.46
DenseNet-201	88.4	86.9	84.7	87.5	0.44
Dual-Stream (DenseNet-201 + ResNet-50)	95.6	93.0	91.9	93.7	0.31

**Fig. 7:**
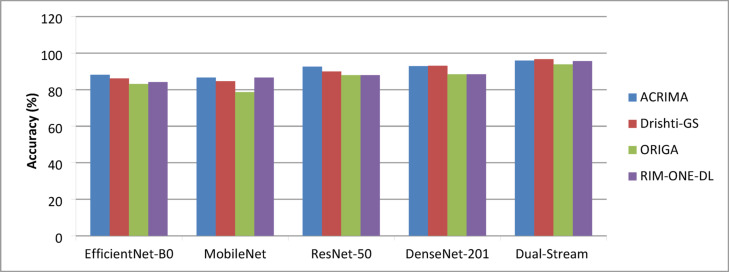
Performance of CNN models across different datasets.

DenseNet-201 and ResNet-50 outperform other single-stream models across all metrics. DenseNet-201 achieved the highest individual performance on the Drishti-Gs dataset, while ResNet-50 maintained strong recall across all datasets, which is critical for minimizing false negatives in clinical screening. Integrating both models within the proposed dual-stream CNN consistently delivered superior results, as illustrated in Fig. 7, achieving up to 96.7% accuracy and 95.9% AUC on the Drishti-Gs dataset, along with a notable reduction in loss values. This performance improvement is attributed to the model’s capacity to jointly exploit both structural and texture-based retinal features. On average, the dual-stream CNN model improved accuracy by 3.5% and recall by 5.2% compared to its individual components. These findings validate the robustness and generalizability of the proposed architecture and reinforce the decision to select DenseNet-201 and ResNet-50 as the core components for subsequent glaucoma detection experiments. These results justify their selection as the backbone of the proposed dual-stream framework, enabling a balanced representation of structural and textural retinal features.

#### Second experiment: assessing the impact of the hybrid data augmentation strategy

In the second experiment, we assess how well the suggested Hybrid Data Augmentation (HDA) approach improves the generalization abilities of deep learning models for glaucoma detection across various clinical datasets. Unlike Traditional Data Augmentation (TDA) methods that rely primarily on basic geometric transformations such as rotations, flips, shifts, zooms, and shearing the HDA strategy introduces two key components designed to better simulate real-world clinical variability:

- Dynamic Gaussian Noise Injection: Noise is adaptively generated based on each image’s brightness and contrast, providing a more realistic emulation of noise patterns encountered in clinical imaging.
- Comprehensive Geometric Transformations: A controlled pipeline combining multiple spatial augmentations to replicate variations in anatomical structures and image acquisition conditions.

This experiment investigates the extent to which the hybrid strategy improves model robustness and performance over TDA, without repeating the baseline (non-augmented) results previously established in the first experiment. To ensure consistency, the same CNN models from the first experiment DenseNet-201 and ResNet-50 were used. In addition, the Dual-Stream model, which integrates both architectures and achieved the best baseline performance, was included to assess its capacity to exploit the HDA approach. All models were trained on the same datasets with the same preprocessing pipeline used in the first experiment, now incorporating data augmentation. The training setup remained: Adam optimizer with a learning rate set at 0.01, a batch size of 32, and 40 epochs for training. The assessment of performance was based on five metrics: accuracy, precision, recall, AUC, and loss, which provided a thorough comparison. The results, summarized in Tables 8, 9, 10, 11, clearly demonstrate the advantages of the HDA approach.

**Table 8:** Performance comparison of single- and dual-stream CNN models using traditional and hybrid data augmentation on the ACRIMA Dataset.

CNN Model	Augmentation type	Accuracy (%)	Precision (%)	Recall (%)	AUC (%)	Loss
ResNet-50	TDA	94.1	92.0	91.2	93.8	0.35
	HDA	95.6	93.4	92.0	93.5	0.32
DenseNet-201	TDA	93.7	91.9	90.4	92.0	0.37
	HDA	95.9	93.7	92.4	93.8	0.30
Dual-Stream (DenseNet-201 + ResNet-50)	TDA	96.3	94.5	93.9	95.2	0.32
	HDA	97.8	96.0	94.7	96.4	0.23

**Table 9:** Performance comparison of single- and dual-stream CNN models using traditional and hybrid data augmentation on the Drishti-Gs Dataset.

CNN Model	Augmentation type	Accuracy (%)	Precision (%)	Recall (%)	AUC (%)	Loss
ResNet-50	TDA	94.4	92.3	90.9	92.8	0.33
	HDA	95.2	93.1	91.7	93.5	0.30
DenseNet-201	TDA	95.7	93.9	91.9	93.7	0.34
	HDA	96.2	94.1	92.8	95.4	0.30
Dual-Stream (DenseNet-201 + ResNet-50)	TDA	97.4	95.4	93.6	95.8	0.25
	HDA	98.3	96.7	95.2	97.3	0.20

**Table 10:** Performance comparison of single- and dual-stream CNN models using traditional and hybrid data augmentation on the ORIGA Dataset.

CNN Model	Augmentation type	Accuracy (%)	Precision (%)	Recall (%)	AUC (%)	Loss
ResNet-50	TDA	93.9	91.5	90.2	91.7	0.37
	HDA	94.4	92.2	91.5	92.5	0.32
DenseNet-201	TDA	94.1	91.8	90.6	92.2	0.36
	HDA	94.7	93.1	91.4	93.4	0.32
Dual-Stream (DenseNet-201 + ResNet-50)	TDA	95.4	94.0	92.5	94.3	0.33
	HDA	96.8	95.2	93.7	95.4	0.30

**Table 11:** Performance comparison of single- and dual-stream CNN models using traditional and hybrid data augmentation on the RIM-ONE-DL Dataset.

CNN Model	Augmentation type	Accuracy (%)	Precision (%)	Recall (%)	AUC (%)	Loss
ResNet-50	TDA	94.0	91.7	90.6	91.9	0.36
	HDA	95.2	92.0	91.8	92.8	0.30
DenseNet-201	TDA	94.1	91.9	90.3	92.4	0.34
	HDA	95.7	93.3	92.6	93.7	0.30
Dual-Stream (DenseNet-201 + ResNet-50)	TDA	96.4	94.8	92.9	95.1	0.28
	HDA	97.3	96.5	94.3	96.8	0.25

Across all datasets and models, HDA consistently improved performance particularly in recall and AUC, which are critical in clinical applications to minimize false negatives. For example: On the ACRIMA dataset, the Dual-Stream model’s accuracy improved from 96.3% to 97.8% and AUC from 90.3% to 93.8%. On Drishti-Gs, accuracy increased from 97.4% to 98.3%, and recall from 92.9% to 94.5%. Similar performance gains were observed on ORIGA and RIM-ONE-DL, with consistent improvements across all evaluation metrics and noticeable reductions in loss values, indicating better model convergence. These findings highlight the value of the Hybrid Data Augmentation strategy in improving generalization across diverse clinical datasets. The most significant improvements were observed in the Dual-Stream architecture, suggesting that it is particularly well-suited to benefit from the increased variability and complexity introduced by HDA. In conclusion, the proposed HDA method significantly enhances the robustness and clinical relevance of deep learning-based glaucoma detection systems. It enables more accurate identification of true glaucoma cases, reduces false negatives, and ensures consistent performance across heterogeneous data sources making it a critical component of the proposed framework.

#### Third experiment: evaluating the impact of lightweight channel-wise attention mechanism

In the third experiment, the objective is to evaluate the effect of integrating a lightweight channel-wise attention mechanism into the glaucoma detection framework. Specifically, this experiment compares the performance of single-stream models (ResNet-50 and DenseNet-121), each augmented with lightweight channel-wise attention, against the proposed dual-stream architecture, which also incorporates the same attention mechanism. The aim is to demonstrate how lightweight channel-wise attention can enhance the discrimination of salient features, improve the overall quality of feature representation, and boost the model’s generalization capability across different datasets. For consistency and fair comparison, the same datasets (ACRIMA, Drishti-Gs, ORIGA, and RIM-ONE-DL), preprocessing pipeline, and HDA strategy from the second experiment were employed. The training protocol was also kept constant: Adam optimizer with a learning rate of 0.01, batch size of 32, and 40 epochs. Evaluation metrics included accuracy, precision, recall, AUC, and loss, providing a comprehensive assessment of the models’ performance.

**Fig. 8:**
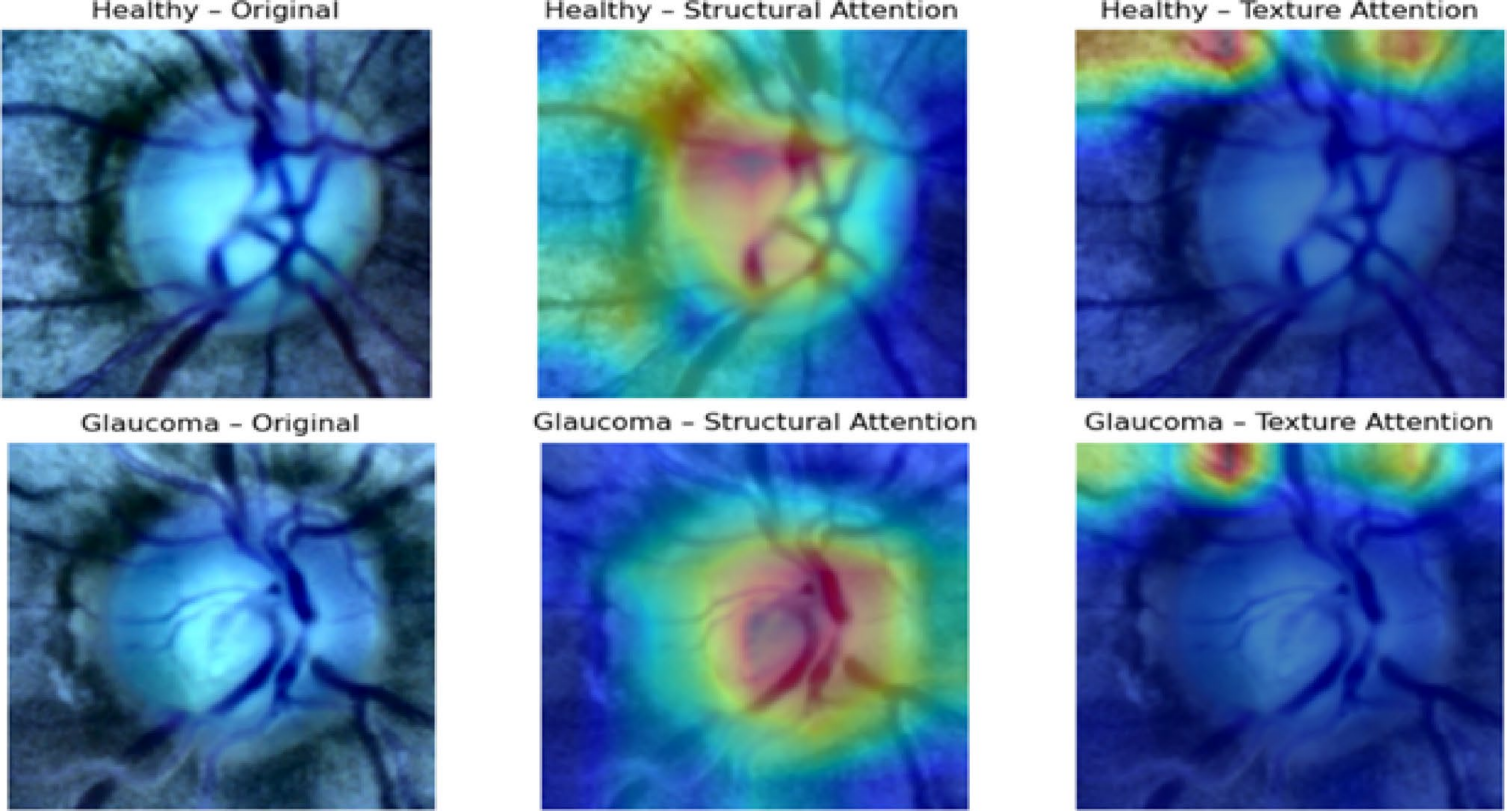
Attention maps for selected examples from the Drishti-Gs dataset.

The results, summarized in Tables 12, 13, 14, 15, demonstrate consistent and measurable improvements over the outcomes of Experiment 2 (HDA-only). Across all datasets, the integration of lightweight channel-wise attention further boosted performance, particularly in recall and AUC metrics crucial for minimizing false negatives in glaucoma detection. For instance, on the ACRIMA dataset, the Dual-Stream model’s accuracy increased from 97.8% (with HDA only) to 98.4% with attention, recall improved from 94.7% to 97.9%, and AUC rose from 96.4% to 98.1%, while the loss decreased from 0.23 to 0.11. On Drishti-Gs, accuracy improved from 98.3% to 98.7%, recall from 95.2% to 97.8%, and AUC from 97.3% to 98.2%, with loss halved from 0.20 to 0.10. Similarly, on ORIGA, accuracy rose from 96.8% to 97.6%, recall from 93.7% to 97.5%, and AUC from 95.4% to 97.4%, with a loss reduction from 0.30 to 0.21. On RIM-ONE-DL, accuracy improved from 97.3% to 97.9% and recall from 94.3% to 97.7%, while loss declined from 0.25 to 0.16. The attention maps in Fig. 8 are shown for selected representative images from the Drishti-Gs dataset for clarity. Similar patterns of attention, highlighting clinically relevant regions of the retina, were consistently observed across the other datasets. This confirms that the behavior illustrated is representative of the model’s overall focus and interpretability.

**Table 12:** Performance of models with channel-wise attention on the ACRIMA Dataset.

CNN Model	Accuracy (%)	Precision (%)	Recall (%)	AUC (%)	loss
ResNet-50 + Attention	96.9	95.3	94.8	95.5	0.25
DenseNet-201 + Attention	97.0	96.3	95.6	96.5	0.22
Dual-Stream + Attention	98.4	97.7	97.9	98.1	0.11

**Table 13:** Performance of models with channel-wise attention on the Drishti-Gs Dataset.

CNN Model	Accuracy (%)	Precision (%)	Recall (%)	AUC (%)	loss
ResNet-50 + Attention	96.6	95.7	95.2	96.0	0.27
DenseNet-201 + Attention	97.4	96.5	95.2	96.5	0.23
Dual-Stream + Attention	98.7	97.5	97.8	98.2	0.10

**Table 14:** Performance of models with channel-wise attention on the ORIGA Dataset.

CNN Model	Accuracy (%)	Precision (%)	Recall (%)	AUC (%)	Loss
ResNet-50 + Attention	96.0	95.2	95.0	94.8	0.29
DenseNet-201 + Attention	96.7	95.8	95.6	95.3	0.23
Dual-Stream + Attention	97.6	97.5	97.5	97.4	0.20

**Table 15:** Performance of models with channel-wise attention on the RIM-ONE-DL Dataset.

CNN Model	Accuracy (%)	Precision (%)	Recall (%)	AUC (%)	Loss
ResNet-50 + Attention	96.4	96.0	95.3	96.3	0.29
DenseNet-201 + Attention	96.8	95.8	95.5	96.7	0.26
Dual-Stream + Attention	97.9	97.3	97.7	97.8	0.16

These results demonstrate that combining HDA with lightweight channel-wise attention in a dual-stream model enhances stability, generalization, and effectively integrates structural and texture features for reliable glaucoma detection.

#### Fourth experiment: evaluating the effectiveness of the improved Manta Ray Foraging Optimization (IMRFO) algorithm in hyperparameter tuning:

In this experiment, the impact of the IMRFO algorithm on hyperparameter tuning is evaluated to enhance the performance of the proposed framework. The IMRFO algorithm, which integrates MRFO with PCOBL, is employed to enable a more efficient and adaptive search for optimal hyperparameter configurations. Key hyperparameters are dynamically fine-tuned by IMRFO based on validation performance, ensuring a balanced trade-off between exploration and exploitation to avoid suboptimal local minima. The optimized hyperparameters include data augmentation settings such as rotation angle, horizontal and vertical shift ranges, zoom factor, and shear intensity as well as transfer learning parameters, including the optimizer type, learning rate, batch size, and the proportion of trainable layers in DenseNet-121 and ResNet-50. To systematically assess the effectiveness of IMRFO, its performance is compared against various other optimization approaches.

- **Proposed Model with IMRFO Optimization:** representing the complete framework after hyperparameter tuning with the proposed IMRFO algorithm.
- **Proposed Model with MRFO Optimization:** using the standard MRFO algorithm before incorporating the PCOBL enhancement.
- **Proposed Model with Other Metaheuristic Optimizers:** benchmarking IMRFO against widely used techniques such as Particle Swarm Optimization (PSO), Genetic Algorithm (GA), and Grey Wolf Optimizer (GWO).
- **Proposed Dual-Stream Model from the Previous Experiment:** serving as the baseline, this model integrates Channel-Wise Attention and Hybrid Data Augmentation but lacks any hyperparameter optimization.

**Fig. 9:**
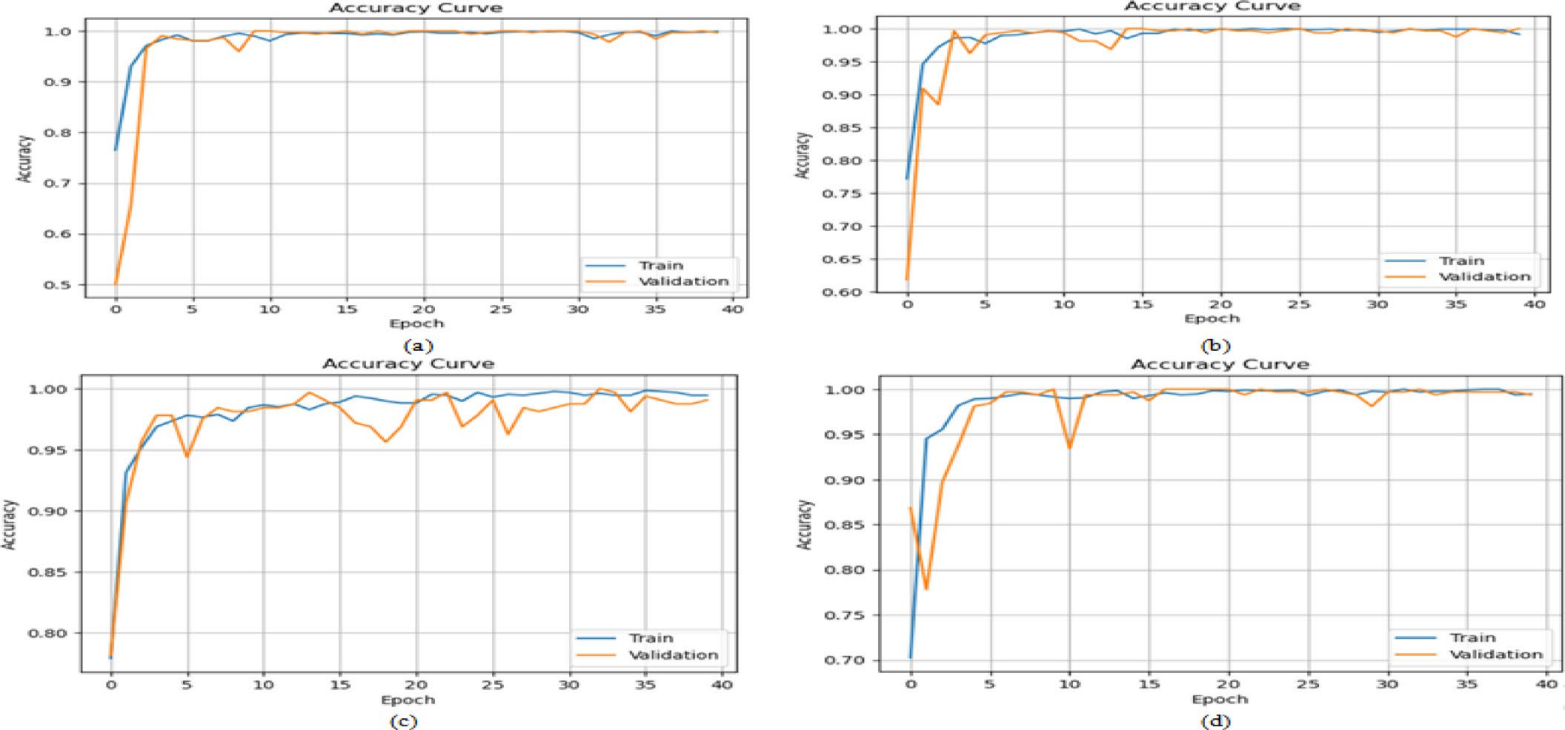
Training and validation accuracy curves of the proposed model across four datasets: (**a**) ACRIMA, (**b**) Drishti-Gs, (**c**) ORIGA, (**d**) RIM-ONE-DL.

**Fig. 10:**
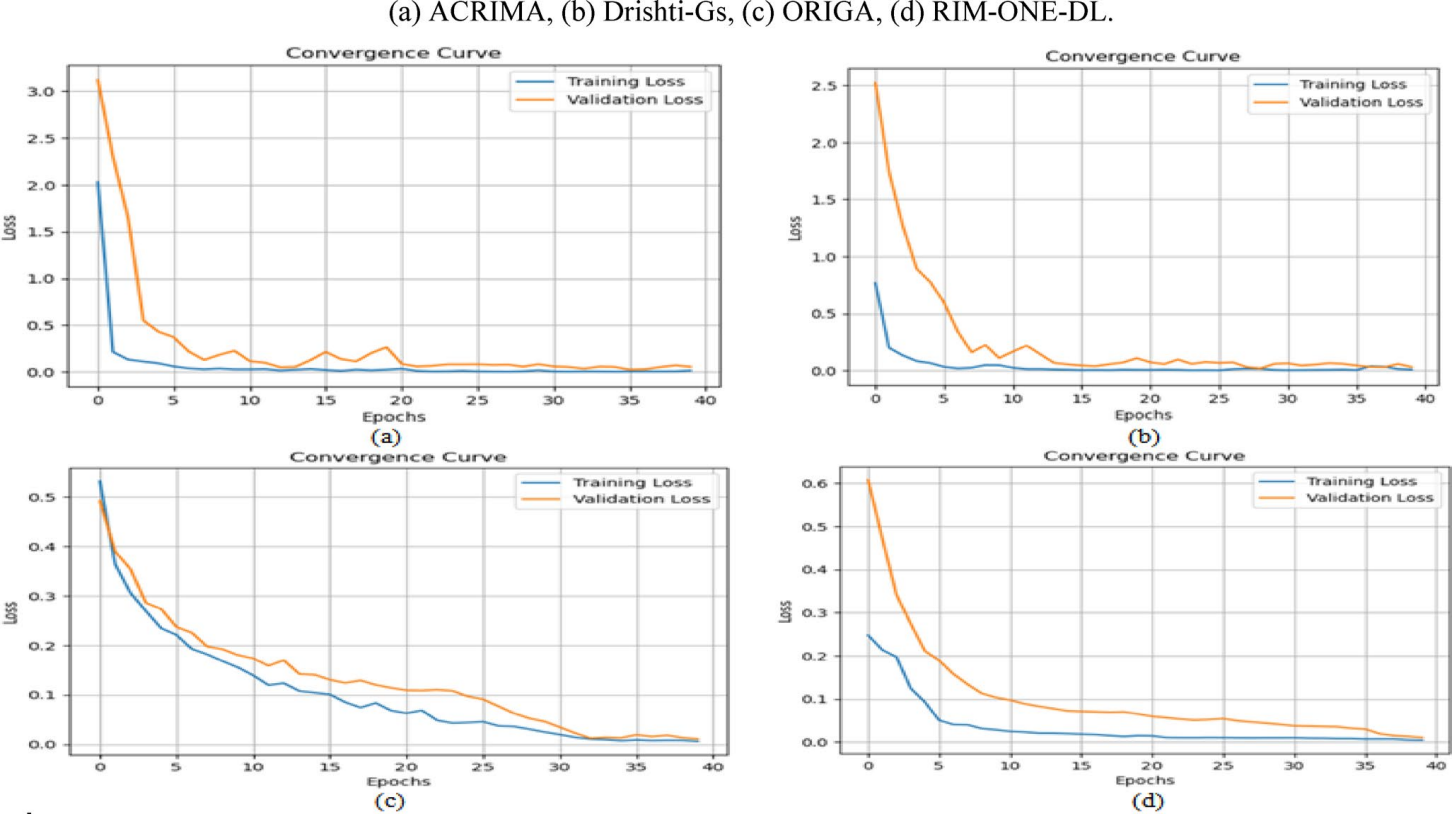
Training and validation convergence curves of the proposed model across four datasets: (**a**) ACRIMA, (**b**) Drishti-Gs, (**c**) ORIGA, (**d**) RIM-ONE-DL.

**Fig. 11:**
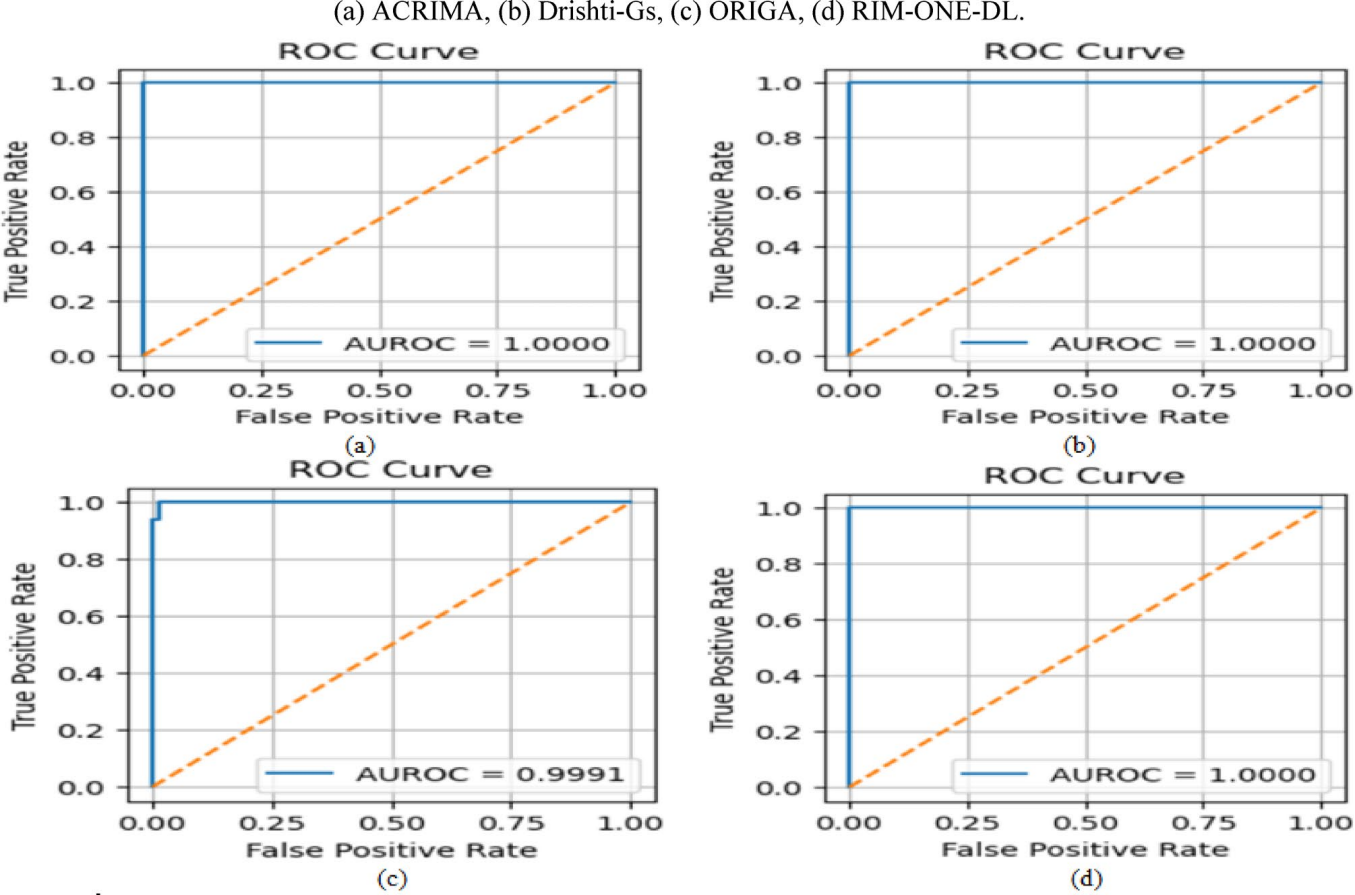
AUROC Curves of the Proposed Model on four test datasets: (**a**) ACRIMA, (**b**) Drishti-Gs, (**c**) ORIGA, (**d**) RIM-ONE-DL.

**Fig. 12:**
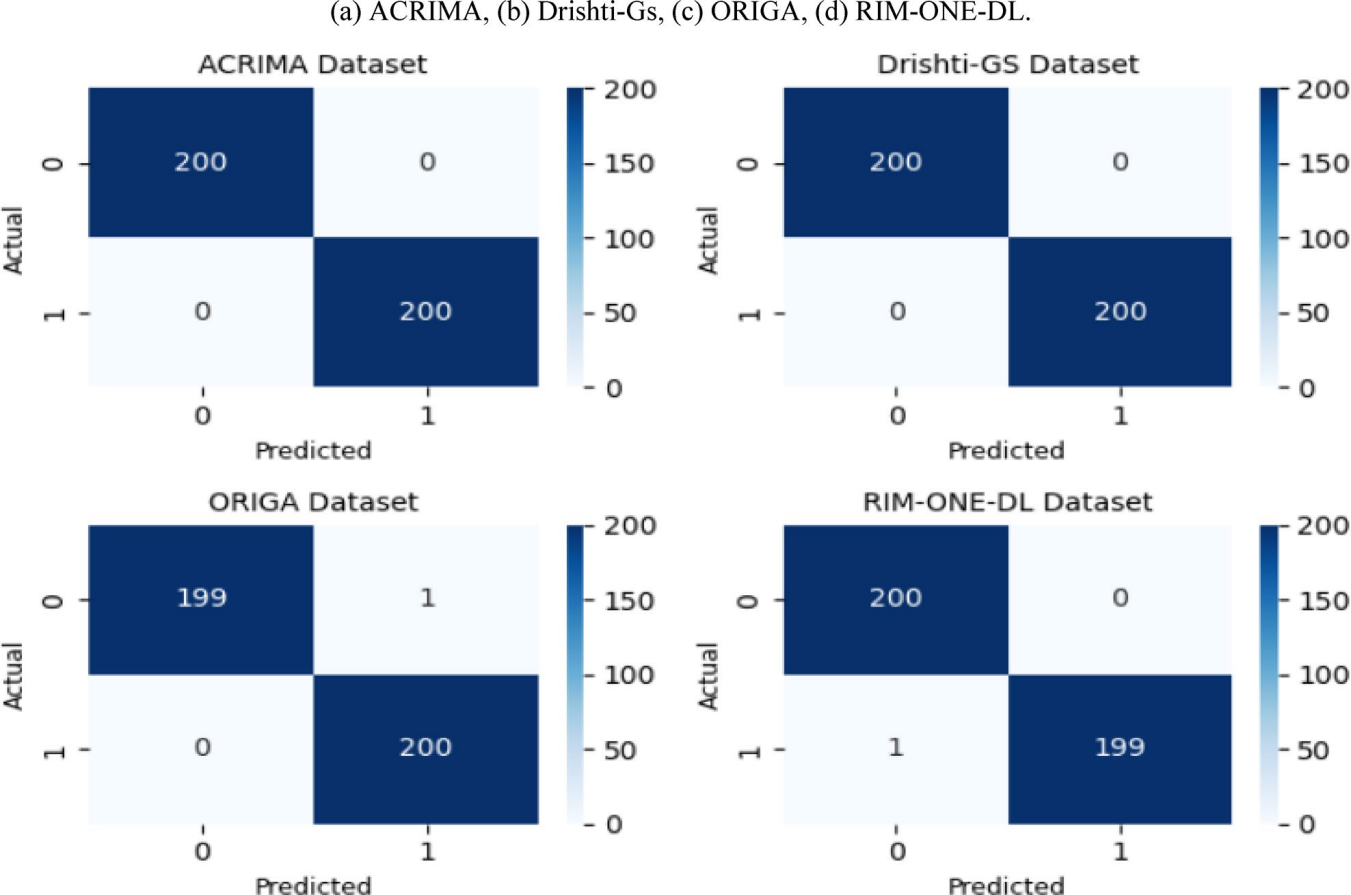
Confusion matrices of the proposed model across different glaucoma.

Each model is trained on the ACRIMA, Drishti-Gs, ORIGA, and RIM-ONE-DL datasets while maintaining the same preprocessing and data augmentation pipeline established in previous experiments. The results presented in Tables 16, 17, 18, 19 clearly demonstrate that the proposed model, optimized using IMRFO, consistently outperforms other optimization techniques across all datasets. Notably, it yields substantial improvements in recall and AUC two metrics of paramount importance in glaucoma detection achieving near-perfect or perfect scores on specific datasets (e.g., ACRIMA and Drishti-Gs). These results reflect dataset-specific outcomes rather than universal performance and are intended to highlight the effectiveness of the proposed optimization strategy. For instance, on the ACRIMA dataset, recall increased from 96.1% (Third Experiment) to 100%, and AUC improved from 97.2% to 100%, with a corresponding drop in loss from 0.12 to 0.003. Comparable performance gains were observed across the Drishti-Gs, ORIGA, and RIM-ONE-DL datasets. These results underscore IMRFO’s effectiveness in fine-tuning hyperparameters, leading to more stable convergence, enhanced generalization, and superior diagnostic performance (Figs. 9, 10, 11, 12).

**Table 16:** Performance comparison of the proposed model with different optimization methods on the ACRIMA Dataset.

CNN Model	Accuracy (%)	Precision (%)	Recall (%)	AUC (%)	loss
Proposed Model without optimization	98.4	96.7	96.1	97.2	0.12
Proposed Model with PSO	98.0	98.8	97.0	98.4	0.09
Proposed Model with GA	98.6	98.9	97.4	99.2	0.03
Proposed Model with GWO	97.8	97.4	96.8	98.2	0.07
Proposed Model with MRFO	98.9	99.2	98.3	99.5	0.04
Proposed Model with IMRFO	100.0	100.0	100.0	100.0	0.003

**Table 17:** Performance comparison of the proposed model with different optimization methods on the Drishti-Gs Dataset.

CNN Model	Accuracy (%)	Precision (%)	Recall (%)	AUC (%)	loss
Proposed Model without optimization	98.7	97.5	96.8	98.2	0.10
Proposed Model with PSO	98.8	97.7	97.5	98.5	0.09
Proposed Model with GA	97.5	96.3	95.7	96.9	0.16
Proposed Model with GWO	97.9	96.6	95.9	97.3	0.12
Proposed Model with MRFO	99.2	98.9	98.6	99.6	0.04
Proposed Model with IMRFO	100.0	100.0	100.0	99.8	0.001

**Table 18:** Performance comparison of the proposed model with different optimization methods on the ORIGA Dataset.

CNN Model	Accuracy (%)	Precision (%)	Recall (%)	AUC (%)	loss
Proposed Model without optimization	97.6	96.5	96.0	96.9	0.21
Proposed Model with PSO	97.9	96.5	96.8	98.3	0.13
Proposed Model with GA	97.0	95.3	95.9	97.1	0.19
Proposed Model with GWO	97.4	95.6	95.3	98.0	0.15
Proposed Model with MRFO	98.4	97.2	97.6	98.7	0.11
Proposed Model with IMRFO	99.7	99.8	99.3	99.5	0.01

**Table 19:** Performance comparison of the proposed model with different optimization methods on the RIM-ONE-DL Dataset.

CNN Model	Accuracy (%)	Precision (%)	Recall (%)	AUC (%)	loss
Proposed Model without optimization	97.9	96.3	95.7	96.8	0.16
Proposed Model with PSO	97.7	97.4	98.4	99.4	0.07
Proposed Model with GA	96.8	97.6	95.9	98.0	0.19
Proposed Model with GWO	98.5	97.8	97.4	98.8	0.06
Proposed Model with MRFO	99.0	98.9	98.3	99.5	0.04
Proposed Model with IMRFO	99.9	99.7	99.5	99.7	0.006

The optimal hyperparameter values discovered by IMRFO for each dataset are summarized in Table 20. These values reflect the adaptive tuning process performed by the optimizer, where parameters such as rotation, shift, zoom, and shear were tailored to the specific distribution and variability of each dataset. Moreover, transfer learning settings such as optimizer choice, learning rate, batch size, and the proportion of trainable layers in DenseNet121 and ResNet50 were also fine-tuned to maximize learning efficiency and generalization. This detailed tuning process played a pivotal role in improving model performance across all evaluation metrics, particularly recall and AUC, as discussed in the previous tables. The consistency of these results across diverse datasets underscores the robustness and flexibility of the IMRFO-driven optimization pipeline.

**Table 20:** Optimal hyperparameter values identified by IMRFO for each dataset.

ACRIMA	Drishti-Gs	ORIGA	RIM-ONE-DL	Hyperparameter
17	29	10	15	Rotation
0.1	0.1	0.1	0.1`	Width Shift
0.2	0.1	0.1	0.1	Height Shift
0.2	0.1	0.1	0.1	Zoom
0.1	0.2	0.2	0.1	Shear
True	False	True	True	Horizontal Flip
True	True	True	True	Vertical Flip
Adam	Adam	Adam	Adam	Optimizer
32	32	32	32	Batch Size
16	24	13	5	TL Learn Ratio
0.01	0.01	0.001	0.01	Learning rate

#### Ablation study

An ablation study is conducted to assess the contribution of each major component in the proposed framework^13^. This analysis systematically evaluates the incremental effects of HDA, the lightweight channel-wise attention mechanism, and IMRFO optimization on model performance. Components are progressively integrated into the baseline dual-stream architecture, with performance measured using Accuracy, Precision, Recall, and AUC. Table 21 presents the results.

**Table 21:** Ablation study: incremental contribution of HDA, attention mechanism, and IMRFO optimization across datasets.

Dataset	Exp	Dual-stream	HDA	Attention	MRFO	Accuracy (%)	Precision (%)	Recall (%)	AUC (%)
ACRIMA	1st	✓	✗	✗	✗	95.9	92.7	91.2	93.8
	2nd	✓	✓	✗	✗	97.8	96.0	94.7	96.4
	3rd	✓	✓	✓	✗	98.4	97.7	97.9	98.1
	4th	✓	✓	✓	✓	**100.0**	**100.0**	**100.0**	**100.0**
Drishti-Gs	1st	✓	✗	✗	✗	96.7	94.7	93.2	95.9
	2nd	✓	✓	✗	✗	98.3	96.7	95.2	97.3
	3rd	✓	✓	✓	✗	98.7	97.5	97.8	98.2
	4th	✓	✓	✓	✓	**100.0**	**100.0**	**100.0**	**99.8**
ORIGA	1st	✓	✗	✗	✗	93.8	91.7	90.5	92.8
	2nd	✓	✓	✗	✗	96.8	95.2	93.7	95.4
	3rd	✓	✓	✓	✗	97.6	97.5	97.5	97.4
	4th	✓	✓	✓	✓	**99.7**	**99.8**	**99.3**	**99.5**
RIM-ONE-DL	1st	✓	✗	✗	✗	95.6	93.0	91.9	93.7
	2nd	✓	✓	✗	✗	97.3	96.5	94.3	96.8
	3rd	✓	✓	✓	✗	97.9	97.3	97.7	97.8
	4th	✓	✓	✓	✓	**99.9**	**99.7**	**99.5**	**99.7**

Significant values are in [bold].

Results show a consistent improvement with each added component. HDA enhances generalization by mitigating dataset variability, the attention mechanism improves feature refinement with negligible parameter overhead, and IMRFO optimization achieves the highest performance gains by stabilizing convergence during training. Notably, the attention module is lightweight and IMRFO affects only the training phase, ensuring that inference-time complexity remains unchanged. These findings confirm that the cumulative performance improvements are achieved without introducing unnecessary computational burden, thereby justifying the proposed multi-component design.

#### Statistical significance analysis of model components

To verify the statistical significance of the performance improvements, paired t-tests were conducted on four benchmark datasets. The analysis follows the incremental development of the framework, where the dual-stream CNN serves as the baseline from Experiment 1, and HDA, attention mechanism, and IMRFO optimization are sequentially added in Experiments 2–4. Identical data splits were used across all Experiments, enabling paired comparisons. Table 22 presents the Accuracy and AUC values obtained at each experimental stage, showing consistent performance gains with each added component.

**Table 22:** Accuracy and AUC results across experimental stages on four benchmark datasets.

Dataset	Metric	Exp. 1 (Baseline Dual-Stream)	Exp. 2 (+ HDA)	Exp. 3 (+ Attention)	Exp. 4 (Full Model + IMRFO)
ACRIMA	Accuracy	95.9	97.8	98.4	100.0
	AUC	93.8	96.4	98.1	100.0
Drishti-Gs	Accuracy	96.7	98.3	98.7	100.0
	AUC	95.9	97.3	98.2	99.8
ORIGA	Accuracy	93.8	96.8	97.6	99.7
	AUC	92.8	95.4	97.4	99.5
RIM-ONE-DL	Accuracy	95.6	97.3	97.9	99.9
	AUC	93.7	96.8	97.8	99.7

Paired t-tests were applied using Accuracy and AUC as primary metrics, due to their clinical relevance. The p-values indicate the probability that the observed improvements could occur under the null hypothesis (no true difference). Values below 0.05 are considered statistically significant, with smaller p-values representing stronger evidence against the null hypothesis.

Tables 23 and 24 summarize the paired t-test results for Accuracy and AUC, respectively. Each component contributed statistically significant improvements, with the largest gains observed after incorporating IMRFO optimization. Overall, the full framework achieved notable improvements over the baseline (4.07% in Accuracy and 6.10% in AUC).

**Table 23:** Paired t-test results for accuracy across experimental stages.

Comparison pair	Added component	Mean difference (%)	t-statistic	*p*-value
Exp. 2 vs. Exp. 1	+ HDA	1.80	15.59	0.0006
Exp. 3 vs. Exp. 2	+ Attention Mechanism	0.52	5.86	0.0098
Exp. 4 vs. Exp. 3	+ IMRFO Optimization	1.75	10.10	0.0021
Exp. 4 vs. Exp. 1	Full Framework vs. Baseline	4.07	11.53	0.0013

**Table 24:** Paired t-test results for AUC across experimental stages.

Comparison pair	Added component	Mean difference (%)	t-statistic	*p*-value
Exp. 2 vs. Exp. 1	+ HDA	2.55	12.37	0.0011
Exp. 3 vs. Exp. 2	+ Attention Mechanism	1.05	4.89	0.0163
Exp. 4 vs. Exp. 3	+ IMRFO Optimization	2.50	14.53	0.0007
Exp. 4 vs. Exp. 1	Full Framework vs. Baseline	6.10	13.56	0.0008

This analysis confirms that each component contributes a statistically significant improvement in both Accuracy and AUC. The p-values, all well below 0.05, demonstrate that the observed gains are highly unlikely to occur by chance, validating the robustness and effectiveness of the proposed framework.

#### Comparative analysis with state-of-the-art glaucoma detection models

In this experiment, the full proposed model is compared with state-of-the-art models from related studies on glaucoma detection. The objective of this comparison is to demonstrate the competitive advantages of the proposed framework in terms of detection accuracy, robustness, and clinical applicability. As presented in Table 25, the proposed model achieved outstanding performance across all evaluation metrics on the benchmark datasets (ACRIMA, Drishti-Gs, ORIGA, and RIM-ONE-DL). These results indicate a superior ability to correctly identify glaucoma cases, which is especially critical for clinical use, where high sensitivity (recall) and discriminative power (AUC) are essential. Furthermore, the integration of the dual-stream architecture, hybrid data augmentation strategy, multi-scale attention mechanisms, and IMRFO-based hyperparameter optimization has been shown to significantly enhance model generalization and robustness, outperforming existing solutions in the literature.

**Table 25:** Comparison with recent methods.

Authors	Datasets	Accuracy (%)	Precision (%)	Recall (%)	AUC (%)	Loss
Kashyap et al.^29^	RIM-ONE DL	96.90	–	–	–	–
Nawaz et al.^30^	ORIGA	97.60	–	96.30	–	–
	RIM-ONE DL	97.80	–	97.00	–	–
Saha et al.^31^	Local dataset of 6671 images	97.4		97.5	99.3	–
Shoukat et al.^33^	ORIGA	92.59	–	98.39	93.00	–
	RIM-ONE DL	96.15	–	97.85	94.20	–
	DRISHTI-GS	97.03	–	93.75	96.00	–
	G1020	98.48	–	99.30	97.00	–
Velpula et al.^34^	ACRIMA	99.57	–	–	100.00	–
	RIM-ONE	94.95	–	–	97.21	–
	HVD	85.43	–	–	97.03	–
	DRISHTI-GS	90.55	–	–	94.36	–
Muduli et al.^35^	ACRIMA	92.23	–	91.13	–	–
	RIM-ONE	92.31	–	89.39	–	–
	G1020	93.25	–	93.26	–	–
	ORIGA	96.75	–	96.67	–	–
Subha et al.^36^	RIM-ONE	98.67	98.17	98.00	98.10	–
	DRIONS-DB	97.71	97.21	97.35	97.65	–
	HRF	97.22	96.83	96.56	96.81	–
	DRISHTI-GS	97.50	97.30	97.01	97.43	–
Sharma et al.^37^	ORIGA	98.46	–	–	–	–
	G1020	97.80	–	–	–	–
Rangaiah et al.^39^	DRISHTI-GS	99.00	98.64	98.50	–	–
	DRIONS-DB	99.50	98.80	99.00	–	–
	HRF	98.50	98.60	98.04		
Sivakumar et al.^40^	Local dataset of	99.4	–	–	–	–
	2874images					
Proposed model	ACRIMA	100.00	100.00	100.00	100.00	0.003
	DRISHTI-GS	100.00	100.00	100.00	99.80	0.001
	ORIGA	99.70	99.80	99.30	99.50	0.01
	RIM-ONE DL	99.90	99.70	99.50	99.70	0.006

## Conclusion and future work

This study introduced a comprehensive deep learning framework for glaucoma detection, integrating several novel contributions to enhance model performance, generalization, and interpretability. The proposed dual-stream architecture, combining DenseNet-121 and ResNet-50, effectively captures both structural and textural features, leading to superior classification performance compared to single-stream CNN models. Additionally, a hybrid data augmentation strategy was developed, incorporating traditional transformations and dynamic Gaussian noise injection, significantly improving model robustness against real-world variations. To further refine the framework, a lightweight channel-wise attention mechanism was integrated into each stream, amplifying salient features and improving feature representation. Hyperparameter optimization was then conducted using the IMRFO algorithm, which demonstrated superior tuning capabilities compared to standard MRFO and other metaheuristic optimizers. The effectiveness of the proposed framework was validated across four benchmark datasets. On ACRIMA, the model achieved 100.00% in all four metrics, with a loss of 0.003. On Drishti-Gs, the model again reached 100.00% in accuracy, precision, recall, and 99.80% AUC, with a loss of 0.001. For ORIGA, performance remained strong with 99.70% accuracy, 99.80% precision, 99.30% recall, and 99.50% AUC (loss = 0.01). Finally, on RIM-ONE-DL, the model achieved 99.90% accuracy, 99.70% precision, 99.50% recall, and 99.70% AUC (loss = 0.006). The consistent outcomes observed across various datasets highlight the strength, generalization ability, and diagnostic dependability of the proposed model.

Future work will focus on enhancing the explainability of the model through attention-based heatmaps and uncertainty estimation, providing clinicians with interpretable insights. Validation on independent multi-center datasets and feedback from ophthalmologists will be conducted to assess generalization and practical usability. Additionally, incorporating multimodal imaging data, such as 2D fundus photography combined with 3D OCT scans, may improve diagnostic precision by capturing complementary structural and textural information of the optic nerve head^63,64^. Finally, optimizing the framework for lightweight deployment will support real-time glaucoma screening in resource-constrained settings.

## Data Availability

Data is available at: https://www.kaggle.com/datasets/ayush02102001/glaucoma-classification-datasets.
